# Endothelial Function is improved by Inducing Microbial Polyamine Production in the Gut: A Randomized Placebo-Controlled Trial

**DOI:** 10.3390/nu11051188

**Published:** 2019-05-27

**Authors:** Mitsuharu Matsumoto, Yusuke Kitada, Yuji Naito

**Affiliations:** 1 Dairy Science and Technology Institute, Kyodo Milk Industry Co. Ltd., Tokyo 190-0182, Japan; y-kitada@meito.co.jp; 2 Molecular Gastroenterology and Hepatology, Kyoto Prefectural University of Medicine, Kyoto 602-8566, Japan; ynaito@koto.kpu-m.ac.jp

**Keywords:** *Bifidobacterium animalis* subsp. *lactis*, arginine, putrescine, spermidine, endothelial function, intestinal microbiome, autophagy

## Abstract

Recently, it was demonstrated that spermidine-induced autophagy reduces the risk of cardiovascular disease in mice. Intestinal bacteria are a major source of polyamines, including spermidine. We previously reported that the intake of both *Bifidobacterium animalis* subsp. *lactis* (Bifal) and arginine (Arg) increases the production of putrescine, a spermidine precursor, in the gut. Here, we investigated the effects of Bifal and Arg consumption on endothelial function in healthy subjects. Healthy individuals with body mass index (BMI) near the maximum value in the “healthy” range (BMI: 25) (*n* = 44) were provided normal yogurt containing Bifal and Arg (Bifal + Arg YG) or placebo (normal yogurt) for 12 weeks in this randomized, double-blinded, placebo-controlled, parallel-group comparative study. The reactive hyperemia index (RHI), the primary outcome, was measured using endo-peripheral arterial tone (EndoPAT). The change in RHI from week 0 to 12 in the Bifal + Arg YG group was significantly higher than that in the placebo group, indicating that Bifal + Arg YG intake improved endothelial function. At week 12, the concentrations of fecal putrescine and serum putrescine and spermidine in the Bifal + Arg YG group were significantly higher than those in the placebo group. This study suggests that consuming Bifal + Arg YG prevents or reduces the risk of atherosclerosis.

## 1. Introduction

Atherosclerosis, a cardiovascular, inflammatory disease characterized by arterial lumen narrowing owing to plaque formation, is a major cause of death globally [1,2]. Endothelial dysfunction occurs early in atherosclerosis development and is involved in plaque formation and disease progression [3]. Therefore, improving endothelial function might help prevent cardiovascular disease in at-risk individuals.

Polyamines (putrescine, spermidine, and spermine), which are involved in the synthesis and stabilization of nucleic acids and stimulation of cell proliferation and differentiation [4], have become a recent focus in the field of atherosclerosis. Autophagy induction by oral spermidine administration reduces blood pressure and cardiovascular disease risk in wild-type mice and salt-sensitive hypertensive rats [5]. The intracellular production and concentration of polyamines in tissues and organs decrease with age [6]; therefore, older individuals are at higher risk of developing atherosclerosis. Polyamines, especially spermine, inhibit inflammatory activity by suppressing pro-inflammatory cytokine synthesis [7] and leukocyte function-associated antigen-1 [8]. Dietary polyamines have also been found to be negatively associated with cardiovascular disease [9]. Therefore, the intake of exogenous polyamines from foods and intestinal bacterial metabolites might benefit cardiovascular health [10,11]. Most dietary polyamines are absorbed in the small intestine [12], and putrescine and spermidine in the colon are provided by intestinal bacteria [13]. However, high levels of polyamines are observed in tumors [14], and the selective inhibition of ornithine decarboxylase, a key enzyme in polyamine synthesis, is effective against malignancies, including colon cancer [14]. Hence, whether polyamines are harmful or beneficial for health remains controversial.

To verify the effect of microbial polyamines on human health, we developed a reliable method to provide polyamines to the body by regulating intestinal bacterial metabolism [15]. This treatment, which involves arginine (Arg) and *Bifidobacterium animalis* subsp. *lactis* administration, increased colonic luminal putrescine levels, and blood spermidine concentrations [15]. This effect is primarily modulated by a pathway involving the independent metabolic systems of three types of bacteria (hybrid putrescine biosynthesis system): *Escherichia coli* (arginine-dependent acid resistance system), *Enterococcus faecalis* (agmatine deiminase-associated energy production system), and *Bifidobacterium* spp. (acid production system) [16]. Thus, putrescine cannot be stably produced by intestinal bacteria unless both *B. animalis* subsp. *lactis* and free Arg (not the constituent Arg in dietary proteins) exist simultaneously in the intestine. Furthermore, *B. animalis* subsp. *lactis* showed the most potent ability to induce putrescine production among *Bifidobacterium* spp. [16]. While our previous studies focused on how polyamine production can be regulated, the application of this method to modulate autophagy and prevent/treat atherosclerosis was not examined.

Here, we evaluated the effects of putrescine produced by the intestinal microbiome following *B. animalis* subsp. *lactis* and Arg administration on endothelial function during the early stages of atherosclerosis development in a randomized, double-blinded, placebo-controlled, parallel-group comparative study.

## 2. Materials and Methods

### 2.1. Subjects

Recruitment was performed at Sapporo Yurinokai Hospital (Sapporo, Japan) based on the disclosed inclusion criteria during one month (September, 2016), under the management of a coordinator in imeQ (Tokyo, Japan), which is a SMO (Site management organization) company. The inclusion criteria were as follows: non-smokers, age of 30–65 years, and body mass index (BMI) <30. The subjects were requested to complete a questionnaire concerning their medical history. Physical examinations, urinalysis, and blood biochemical analyses were also performed. Of 88 candidates recruited, those with the following criteria were excluded: those taking prescription medicines or with a history of hospitalization, severe digestive disorders, digestive organ surgery, or food allergies. We selected 44 healthy subjects with BMI near the maximum value of the range for “healthy” (BMI: 25) according to the Japan society for the study of obesity standard, because obesity is one of the risk factors associated with atherosclerosis. Furthermore, a BMI of 25–30 is classified as “pre-obese” according to WHO standards and “obesity level 1” according to the Japan society for the study of obesity standard. Therefore, we considered the recruited subjects to be individuals having a higher risk of atherosclerosis among healthy people. The sample size was determined using power analysis by referring to a previous study [17] that evaluated endothelial function using endo-peripheral arterial tone (EndoPAT). In brief, using power analysis, 22 patients were estimated to be required for the study to achieve a minimum treatment effect of 0.2 units in the reactive hyperemia index (RHI), a standard deviation of 0.26, one-sided 5% significance level, and 80% power. Randomization was based on a table of random numbers and stratified by age to either the *Bifidobacterium animalis* subsp. *lactis* (Bifal) + Arg YG group (*n* = 22) or the placebo group (*n* = 22). At the beginning of the study (week 0), the blood triglyceride levels of the subjects were analyzed, and those with values lower than the normal range (50–149 mg/dL), referred by the Daiichi Kishimoto Clinical Test Center (Sapporo, Japan) based on the guideline of hyperlipidemia treatment [18], were excluded. Furthermore, subjects found to have severe occult blood in the urine (>250 erythrocytes/μL), referred by the Japanese Committee for Clinical Laboratory Standards [19], were excluded. Subjects were recruited from September 1, 2016, and the intervention study commenced on September 20–23, 2016, and finished on December 13–16, 2016. In accordance with the Declaration of Helsinki, the purpose and content of the examinations were explained to the participants, and their informed consent was obtained before enrollment in the clinical trial.

### 2.2. Preparation of Placebo Yogurt and Yogurt Containing B. Animalis Subsp. Lactis and Arg (*Bifal* + Arg YG)

We prepared the test meal (Bifal + Arg YG) by adding *B. animalis* subsp. *lactis* and Arg to normal yogurt, because this study did not intend to compare the effects of the intake of only *B. animalis* subsp. *lactis* or Arg, but instead evaluate the effects of a mixture of both. Normal yogurt fermented with *Streptococcus thermophilus* and *Lactobacillus delbrueckii* subsp. *bulgaricus* was used as the placebo yogurt because *S. thermophilus* and *L. delbrueckii* subsp. *bulgaricus* contained in normal yogurt (placebo) die due to stomach acid; these bacteria, therefore, do not function as probiotics in the intestine. Bifal + Arg YG was prepared by adding *B. animalis* subsp. *lactis* LKM512 (1.0 × 10^8^ colony-forming units/g) and 600 mg Arg to 100 g of the placebo yogurt. Bifal + Arg YG and placebo yogurt, which had the same color and taste, were packed separately into unlabeled pots. We prepared Bifal + Arg YG and placebo yogurt once every 2 weeks. The yogurt was provided to subjects within 2 weeks of preparation, and it was confirmed that there was no change in the number of *B. animalis* subsp. *lactis* or the Arg content in the yogurt during that period using yogurts stored in the laboratory’s refrigerator for 2 weeks. Viable counts of *B. animalis* subsp. *lactis* and Arg content in each yogurt were confirmed by cultivation on TOS propionate agar (Yakult Pharmaceutical Industry Co. Ltd, Tokyo, Japan), which is a selective medium for enumerating Bifidobacterium spp. from dairy products, and also by the method shown in Section 2.10.

### 2.3. Experimental Design

This study was a double-blinded, placebo-controlled, parallel-group comparative study. Subjects in the Bifal + Arg YG and placebo group ingested 100 g of yogurt once a day (after lunch) for 12 weeks. Both subjects and observers were blinded to the group allocation throughout the trial period. Double-blinding was achieved by labeling the test yogurts with an identification number only. The primary outcome was the RHI, which was measured via EndoPAT. Secondary outcomes included physical examination, systolic blood pressure, diastolic blood pressure, blood biomarkers, BMI, abdominal circumference, fecal markers, and evaluation of safety. Serum and feces samples were also collected at weeks 0 and 12. The study was performed at Sapporo Yurinokai hospital (Sapporo, Japan). All participants were asked to avoid food containing large amounts of viable bacteria, such as probiotic supplements, fermented milk products, lactic acid bacterial drinks, and fermented soybean (natto), during the experimental period. The study protocol was approved by the Iryouhoujinsyadan Hakusuikai SUDA clinic institutional review board. This study is registered in the University hospital medical information network clinical trials registry (UMIN-CTR) (http://www.umin.ac.jp/ctr/index.htm) under the trial ID UMIN000023857.

### 2.4. EndoPAT

Current evidence indicates that reactive hyperemia peripheral arterial tonometry (RH-PAT), which is a noninvasive technique to assess peripheral microvascular endothelial function by measuring changes in digital pulse volume during reactive hyperemia (RH), has the potential to significantly impact the field of cardiovascular research and prevention of cardiovascular disease with the same accuracy as flow-mediated dilation (FMD), and that it is easier and more operator-independent compared to FMD, although several aspects need to be clarified before its widespread application as a promising technique [20]. Therefore, we considered that the evaluation of endothelial function using this method is reasonable for the research aimed at the prevention of the precursor process during cardiovascular disease development using functional food, such as in the current study. The endothelial functional tests, physical examinations, and urine and blood collection were performed under fasting conditions (≥12 h). Endothelial function was measured with an EndoPAT 2000 system (Itamar Medical, Caesarea, Israel). Fasting subjects were requested to lie on a bed in a quiet room. Subsequently, the peripheral arterial tonometry (PAT) probe was attached to the fingers of both hands of the subject, and they were allowed to rest for 15–20 min. The RHI was determined according to the manual accompanying the EndoPAT 2000 system. Endothelial dysfunction is indicated as RHI <1.67, which was calculated according to Bonetti et al. [21] and has served as a key or reasonable value in several clinical trials [22,23]. However, according to several reports, other RHI values were effective to evaluate the risk of several symptoms related to endothelial function [24,25].

### 2.5. Meal Preparation and Fecal Collection Methods

To eliminate the influence of meal contents on fecal metabolites and accurately determine the polyamine and trimethylamine concentrations produced by the intestinal microbiome, all the subjects were provided identical meals on the day before feces collection. The prepared menu, consisting of 1.5 L water and three meals (1,970 kcal/day), was developed and supervised by a nutritionist (Supplementary Figure S1). The amount of calories of the identical meal provided to all subjects was calculated based on the overview of dietary reference intake for Japanese (2015) published from the Japanese Ministry of Health, Labor, and Welfare [26]. In brief, we used the center value (approximately 2,000 kcal/day) of 2,300 kcal for men and 1,750 kcal for women, calculated based on the standard of calorie intake per day for Japanese who are 30–49 years old (average age of this trial) and have a low physical activity level (working style of the subjects was desk work). Therefore, the amount of calories was slightly lower and higher than the standard for men and women, respectively. However, since this trial was conducted without a limitation of calorie intake during the 12-week test period, we consider that this difference of calories/body weight is within the margin of error. On the day prior to feces collection, the subjects consumed the provided breakfast, lunch (containing hijiki, an indigestible seaweed that is used as a marker during fecal analysis), and dinner without leaving the premises. Consumption of food and drinks other than the provided meals was prohibited. Subjects used feces collection sheets “Nagaseru” (Atleta, Osaka, Japan) for fecal collection to prevent contamination with water and chemicals from the toilet. After defecation on this sheet, the subjects visually confirmed the presence of hijiki, and the fecal portion containing hijiki was collected in fecal sampling tubes.

### 2.6. Extraction of Fecal Bacterial DNA

Extraction of fecal bacterial DNA was performed as described in our previous report [27]. Approximately 20 mg of each fecal sample was suspended in 600 μL of extraction buffer containing 60 mM Tris-HCl, 30 mM EDTA, and 0.8% sodium dodecyl sulfate. The suspension was mixed with 500 μL of TE-saturated phenol, incubated at 70 °C for 10 min in a water bath, and vortexed vigorously with 300 mg of glass beads (diameter, 0.1 mm) for 60 s at 4,000 rpm using a Micro Smash MS-100 homogenization system (Tomy, Tokyo, Japan). Then, 350 μL of each supernatant was collected by centrifugation (20,400× *g*, 5 min), and the DNA was purified using Ethachinmate (Nippon Gene, Tokyo, Japan). Finally, the purified DNA was dissolved in nuclease-free water (Thermo Fisher Scientific, Waltham, MA, USA) and stored at −80 °C.

### 2.7. Construction of the 16S rRNA Gene Amplicon Library and Next-Generation Sequencing

The V1-V2 region of the bacterial 16S rRNA gene was amplified by PCR with fusion primers using the fecal DNA as a template. The forward primer contained an Ion A adapter sequence, followed by a key, barcode, adapter (GT), and 27Fmod primer sequence (5′-AGRGTTTGATYMTGGCTCAG-3′). The reverse primer had an Ion truncated P1 adapter and 338R primer sequence (5′-TGCTGCCTCCCGTAGGAGT-3′) [28]. PCR, DNA purification, emulsion PCR, and sequencing were performed using an Ion PGM system (Thermo Fisher Scientific) according to the manufacturer’s instructions.

### 2.8. Data Processing and Sequence Alignment

Sequence data were obtained in FASTQ format and analyzed using QIIME 1 software [29]. Raw sequences were sorted according to their barcode and priming sites and screened using an average quality score ≥20 to obtain sequences of approximately 300 bp. The trimmed sequences were clustered into operational taxonomic units (OTUs) at the level of 97%, and the most abundant sequence in each OTU was chosen as the representative sequence. The representative sequences were aligned and checked for potential chimeric sequences using the ChimeraSlayer algorithm. Non-chimeric sequences were assigned to a taxon using the RDP classifier ver. 11.5 at a confidence cut-off value of 80% [30]. The data of fecal microbiota analysis have been deposited at the DDBJ sequence read archive [31] under accession number DRA006828.

### 2.9. Real-Time PCR for the Quantitative Determination of B. animalis subsp. lactis Cell Numbers

*B*. *animalis* subsp. *lactis* levels were quantified by real-time PCR, as described in our previous study [32] with a slight modification. Briefly, SYBR Premix Ex Taq II (Takara Bio, Otu, Japan), a forward primer (5′-CCCTTTCCACGGGTCCC-3′) and a reverse primer (5′-AAGGGAAACCGTGTCTCCAC-3′), were used. Real-time PCR was performed using the StepOne real-time PCR system (Applied Biosystems, Waltham, MA, USA).

### 2.10. Determination of Fecal Polyamine Concentration

The fecal polyamine concentration and Arg concentration in yogurt was measured using 6-aminoquinolyl-N-hydroxysuccinimidyl carbamate as a derivatization reagent, as described previously [15]. An ACQUITY ultra performance liquid chromatography system with a fluorescence detector (UPLC FLR) (Waters, Milford, MA, USA) was used for the analysis.

### 2.11. Determination of Serum Polyamine Concentration

Serum polyamine was derivatized with ethyl chloroformate and trifluoroacetic anhydride and quantified by gas chromatography-mass spectrometry (GC-MS), according to the methods described by Chen et al. [33], with some modifications. Briefly, 500 μL of serum was added to 1,7-diaminoheptane (internal standard) to obtain a final concentration of 10 μM, and an equal amount of 20% trichloroacetic acid (TCA) was added to precipitate the protein. Then, 1 mL of diethyl ether was added to the supernatant, and the diethyl ether layer containing TCA was removed. This process was repeated, and the residual diethyl ether was completely removed using a centrifugal concentrator (5305 C, Eppendorf, Hamburg, Germany). Next, 5 M NaOH was added to increase the alkalinity (pH 11 or higher) in the extract, and 1150 μL of diethyl ether containing 50 μL of ethyl chloroformate was added and mixed for 30 min. The diethyl ether layer containing *N*-ethoxycarbonylated polyamine was collected and dried, after which 100 μL of ethyl acetate and 200 μL of trifluoroacetic acid were added and reacted at 75 °C for 1 h for complete trifluoroacetylation. Finally, the mixture was dried, 200 μL of ethyl acetate was added, and the solution was subjected to GC-MS analysis using a GC-MS QP2010 (Shimadzu, Kyoto, Japan) equipped with a ZB-5 capillary column (60 m × 0.25 mm × 0.25 μm) (Phenomenex, Torrance, CA, USA). The GC-MS conditions were as follows: injector temperature, 260 °C; column temperature, 140 °C. The reaction was programmed at a rate of 8 °C/min to 190 °C, 190 °C for 5 min, 20 °C/min to 300 °C, 300 °C for 5 min, followed by 20 °C/min to 320 °C. Helium gas was used as the capillary gas, and the flow rate was 5.5 mL/min. The mass spectrometer was operated in selected ion-monitoring mode, and putrescine, 1,7-diaminoheptane, spermidine, and spermine were quantified with m/z 355, 397, 480, and 609, respectively. It was confirmed that an accurate calibration curve could be prepared by this method (Supplementary Figure S2).

### 2.12. Determination of Fecal Trimethylamine

The fecal trimethylamine concentration was measured as described by our previous report [34], using a GC-MS (GC-MS QP2010) equipped with a Rtx-1 (30 m × 0.32 mm × 4.0 mm film thickness) fused silica capillary column (Restek Corporation, Bellefonte, PA, USA). A carboxen-polydimethylsiloxane (75 μm) SPME fiber (Supelco, Bellefonte, PA, USA) was used for analysis.

### 2.13. Physical Examination, Urinalysis, and Blood Biochemical Analyses

Height, weight, BMI, abdominal girth, systolic blood pressure, diastolic blood pressure, and heart rate were measured on the same day as the EndoPAT test. Blood biochemical analyses and urinalysis were performed at the Daiichi Kishimoto Clinical Test Center (Sapporo, Japan).

### 2.14. Measurement of Serum NO_2_/NO_3_, Tumor Necrosis Factor (TNF)-α, and Interleukin (IL)-1β

We used a NO_2_/NO_3_ Assay Kit-FX (Dojindo, Kumamoto, Japan), Human TNF-α Quantikine HS ELISA Kit (R & D Systems, Minneapolis, MN, USA), and IL-1β Human ELISA Kit, high sensitivity (Thermo Fisher Scientific) for the analysis of NO_2_/NO_3_, TNF-α, and IL-1β, respectively, according to the manufacturers’ instructions.

### 2.15. Evaluation of Safety

During the test period, the subjects recorded their test yogurt intake, physical condition, medicine intake, and defecation symptoms. The subjects consulted a doctor concerning any adverse reactions on the day of their visit. Safety was evaluated on the basis of the incidence and severity of adverse diet-related events experienced throughout the study, and the effects observed in the Bifal + Arg YG and placebo groups were compared.

### 2.16. Statistical Analysis

Data are expressed as the mean ± standard error of the mean (SEM), and the comparison between the Bifal + Arg YG and placebo groups was tested by two-way ANOVA, Student’s *t*-test, or the Mann–Whitney *U* test. Two-way ANOVA was performed using measured values (baseline value and change value) as one factor and groups as the second factor, and then simple main effects analysis with Bonferroni adjustment was performed. Comparisons at week 0 and at week 12 were tested using the paired *t*-test or Wilcoxon’s signed rank test. Smirnov–Grubbs tests were performed to identify and remove the outliers among the measured values obtained from the EndoPAT test, physical examination, urinalysis, blood biochemical analyses, and measurement of serum polyamines, NO_2_/NO_3_, TNF-α, and IL-1β (outliers are shown in Supplementary Figure S3). Two-way ANOVA, Student’s *t*-test, and the Mann–Whitney *U* test were performed using SPSS ver. 22 (IBM, Armonk, NY, USA). All other statistical analyses were performed using R statistical software ver. 3.4.2 or SPSS ver. 22.

## 3. Results

### 3.1. Subjects

**Figure 1:**
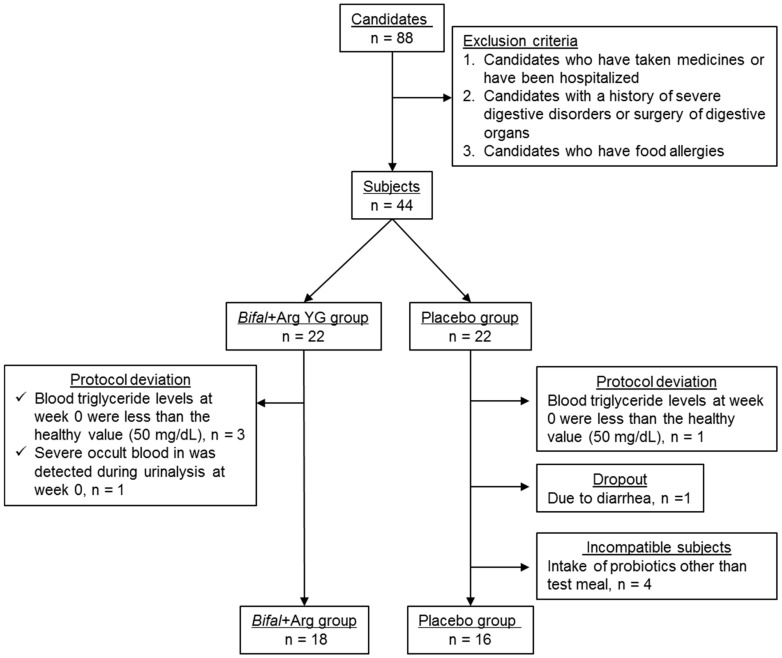
Method followed for subject selection.

In the urinalysis and blood biochemical analyses conducted at week 0, one subject in the Bifal + Arg YG group had severe occult blood in the urine (>250 erythrocytes/μL), while three had serum triglyceride concentrations below the reference value (50 mg/dL). These subjects, along with one subject in the placebo group with similarly low serum triglycerides, were excluded from further study. During the clinical test, one subject in the placebo group experienced diarrhea and was excluded, as advised by the doctor. At week 12, four subjects in the placebo group had high counts (≥3.2 × 10^7^ cells/g of feces) of *B. animalis* subsp. *lactis*, which is not a common commensal bacterium in the human gut [35,36]; therefore, these subjects were also excluded. The remaining subjects in the Bifal + Arg YG group (*n* = 18; 10 men and 8 women; average age, 45.5 years) and placebo group (*n* = 16; 9 men and 7 women; average age, 44.0 years) were retained for further analysis (Figure 1).

### 3.2. RHI

**Figure 2:**
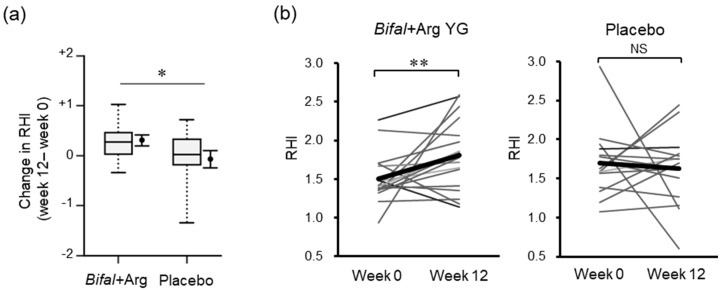
Effect of the consumption of yogurt containing *B. animalis* subsp. *lactis* and Arg (Bifal + Arg YG) on the reactive hyperemia index (RHI). Boxplots represent the 5th percentile, 95th percentile, interquartile range (25–75%), and median. The black circle and error bars on the side of the boxplots represent the average value and SEM, respectively. (**a**) Change in RHI during the clinical test period (* *p* < 0.05, baseline × change interaction *p* = 0.023 by two-way ANOVA). *n* = 18 in the Bifal + Arg YG group and *n* = 15 in the placebo group (one outlier in the placebo group at week 12 was detected; Supplementary Figure S3). (**b**) Change in RHI within each group. The bold line represents the average value. ** *p* < 0.01 by paired *t*-test. NS: Not significant. *n* = 18 in the Bifal + Arg YG group and *n* = 15 in the placebo group (one outlier in the placebo group at week was detected; Supplementary Figure S3).

After the completion of the 12-week period, the change in the RHI in the Bifal + Arg YG group (+0.31 ± 0.12) was significantly higher than that in the placebo group (−0.07 ± 0.18) (*p* < 0.05, baseline × change interaction *p* = 0.023 by two-way ANOVA, Figure 2a). However, the RHI level at the baseline did not differ between the Bifal + Arg YG group and placebo group (*p* = 0.174, Student’s *t*-test) (Table 1). Furthermore, the RHI increased significantly (*p* < 0.01 by paired *t*-test) in the Bifal + Arg YG group but not in the placebo group (Figure 2b, Table 1, Supplementary Table S1).

### 3.3. Systolic Blood Pressure and Diastolic Blood Pressure

The systolic blood pressure at week 12 in the Bifal + Arg YG group (120.2 ± 2.8 mmHg) tended to be lower than that in the placebo group (125.3 ± 2.5 mmHg; *p* = 0.097 by Student’s *t*-test), although there was no difference between both the groups at week 0 (Table 1). The diastolic blood pressure at week 12 in the Bifal + Arg YG group (75.1 ± 2.2 mmHg) tended to be lower than that in the placebo group (78.9 ± 1.9 mmHg; *p* = 0.097 by Student’s *t*-test), although there was no difference between both the groups at week 0 (Table 1, Supplementary Table S1).

### 3.4. Physical Parameters, Urinalysis, and Blood Biochemical Analyses

**Figure 3:**
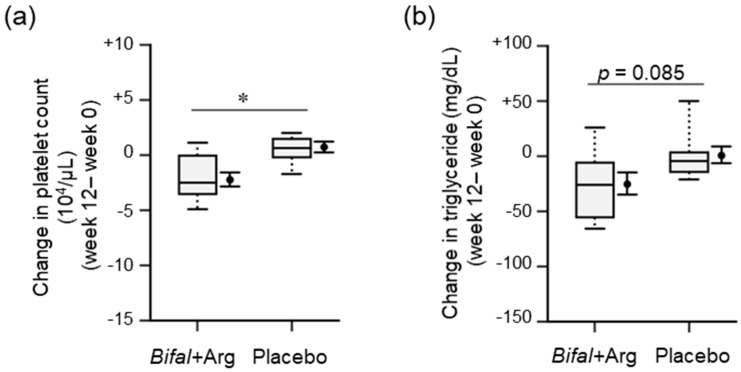
Effect of the consumption of yogurt containing *B. animalis* subsp. *lactis* and Arg (Bifal + Arg YG) on platelet count and blood triglyceride. Boxplots represent the 5th percentile, 95th percentile, interquartile range (25–75%), and median. The black circle and error bars on the side of the boxplots represent the average value and SEM, respectively. (**a**) Change in platelet count during the clinical test period. * *p* < 0.05, baseline × change interaction *p* = 0.111 by two-way ANOVA). *n* = 18 in the Bifal + Arg YG group and *n* = 16 in the placebo group. (**b**) Change in triglyceride level during the clinical test period (*p* = 0.085, baseline × change interaction *p* = 0.061 by two-way ANOVA). *n* = 18 in the Bifal + Arg YG group and *n* = 14 in the placebo group (two outliers in the placebo group were detected; Supplementary Figure S3).

We found several significant differences and relative changes during physical examinations (Table 1, Supplementary Table S1), blood biochemical analyses (Table 2, Supplementary Table S1), and urinalysis (Supplementary Table S1). The change in serum platelet concentration during the clinical test in the Bifal + Arg YG group ((−2.2 ± 0.6) × 10^4^/μL) was significantly lower than that in the placebo group ((+0.7 ± 0.5) × 10^4^/μL; *p* < 0.05, baseline × change interaction *p* = 0.111 by two-way ANOVA, Figure 3a). The platelet concentration at week 12 in the Bifal + Arg YG group ((25.6 ± 1.1) × 10^4^/μL)) was significantly lower than that in the placebo group ((28.4 ± 1.0) × 10^4^/μL); *p* < 0.05 by Student’s *t*-test), although there was no difference between both groups at week 0 (Table 1). The platelet concentration decreased significantly (*p* < 0.01 by paired *t*-test) in the Bifal + Arg YG group but not in the placebo group (Table 1). Furthermore, the change in serum triglyceride concentration during the clinical test in the Bifal + Arg YG group (−25.4 ± 10.5 mg/dL) tended to be lower than that in the placebo group (+0.6 ± 8.0 mg/dL) (*p* = 0.085, baseline × change interaction *p* = 0.061 by two-way ANOVA; Figure 3b). At week 12, the Bifal + Arg YG group also showed a significant decrease in the serum triglyceride (*p* < 0.05 by paired *t*-test) and a significant increase in the serum high-density lipoprotein (HDL) cholesterol levels (*p* < 0.05 by paired *t*-test) compared with those observed at week 0 (Table 1). However, no such changes were observed in the placebo group. No change in BMI or difference in BMI between the two groups was found. Although abdominal circumference in both groups was increased (*p* < 0.01), there was no difference in the change in total cholesterol between the groups.

### 3.5. Serum NO_2_/NO_3_, TNF-α, IL-1β, and Fecal Trimethylamine Concentrations

At week 12, the serum NO_2_/NO_3_ concentrations between the Bifal + Arg YG and placebo groups were not significantly different (21.3 ± 1.5 μM and 24.5 ± 2.1 μM, respectively; Table 1). Similarly, at week 12, the serum TNF-α and IL-1β concentrations in the Bifal + Arg YG group (0.55 ± 0.03 pg/mL and 0.046 ± 0.010 pg/mL, respectively; Table 1) were not significantly different from those in the placebo group (0.62 ± 0.05 pg/mL and 0.039 ± 0.007 pg/mL, respectively; Table 1). The fecal trimethylamine concentrations between the Bifal + Arg YG and placebo groups were also not significantly different (118.0 ± 25.2 μM and 97.9 ± 16.6 μM, respectively) at week 12.

### 3.6. Polyamine Concentration in Feces and Serum

**Figure 4:**
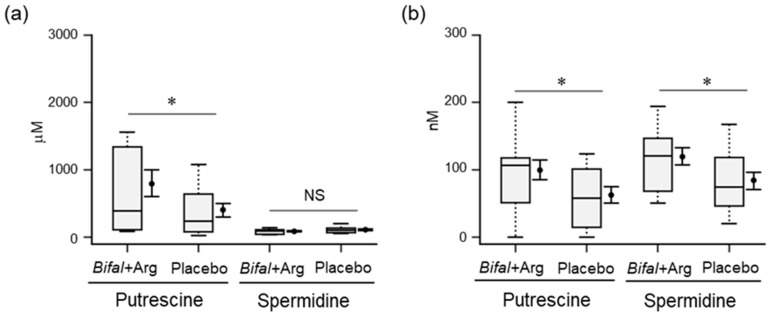
Effect of the consumption of yogurt containing *B. animalis* subsp. *lactis* and Arg (Bifal + Arg YG) on the concentration of fecal and serum polyamines. Boxplots represent the 5th percentile, 95th percentile, interquartile range (25–75%), and median. The black circle and error bars on the side of the boxplots represent the average value and SEM, respectively. (**a**) Fecal putrescine and spermidine concentrations at week 12. * *p* < 0.05, Mann–Whitney *U* test. *n* = 17 in the Bifal + Arg YG group and n = 16 in the placebo group. One subject in the Bifal + Arg YG group did not submit feces; therefore, it was regarded as a missing value. (**b**) Serum putrescine and spermidine concentrations at week 12. * *p* < 0.05, Student’s *t*-test. In the case of serum putrescine, *n* = 17 in the Bifal + Arg YG group and *n* = 16 in the placebo group (one outlier in the Bifal + Arg YG group was detected; Supplementary Figure S3). In the case of serum spermidine, *n* = 17 in the Bifal + Arg YG group and *n* = 16 in the placebo group (one outlier in the Bifal + Arg YG group were detected; Supplementary Figure S3).

At week 12, the fecal and serum putrescine concentrations in the Bifal + Arg YG group (792.1 ± 199.0 μM and 99.5 ± 15.6 nM, respectively) were significantly higher than those in the placebo group (406.4 ± 102.2 μM and 62.6 ± 13.0 nM, respectively; *p* < 0.05 by Mann–Whitney *U* test; Figure 4a,b). Furthermore, while the fecal spermidine concentrations in the Bifal + Arg YG and placebo groups were not significantly different at week 12 compared with that at week 0 (Figure 4a), the serum spermidine concentration in the Bifal + Arg YG group (119.4 ± 13.6 nM) was significantly higher than that in the placebo group (84.4 ± 13.0 nM; *p* < 0.05 by Student’s *t*-test; Figure 4b). In most subjects, neither fecal nor serum spermine was detected (detection limit: 2.0 µM). 

### 3.7. Fecal Microbiota

**Figure 5:**
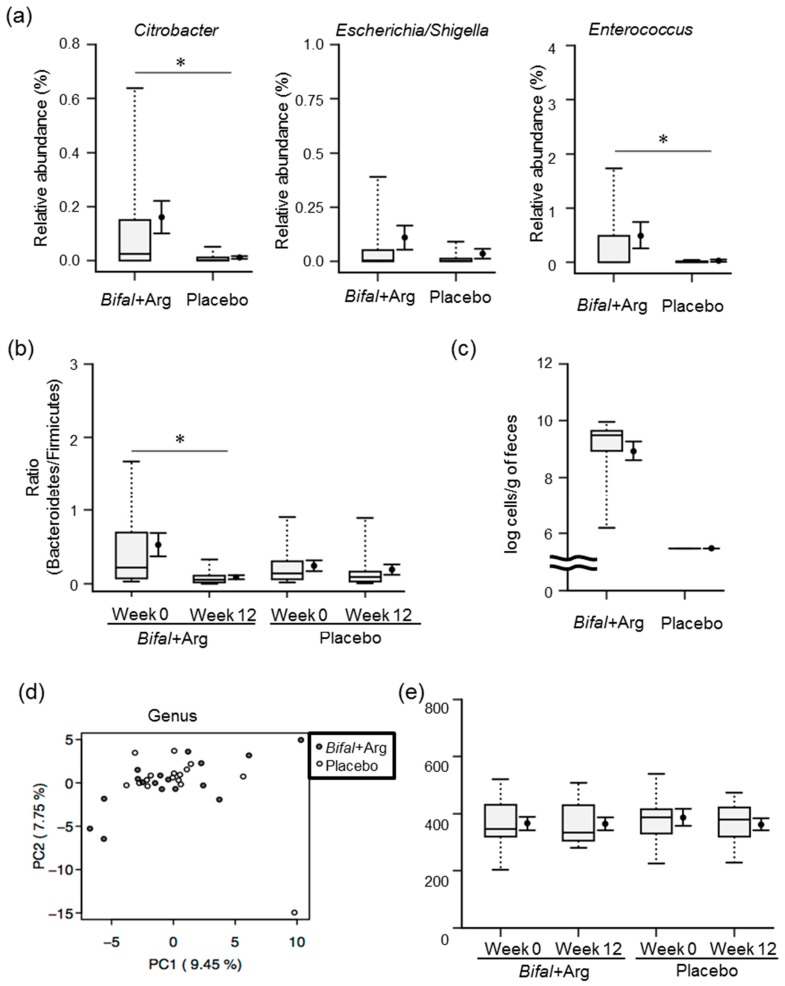
Effect of the consumption of yogurt containing *B. animalis* subsp. *lactis* and Arg (Bifal + Arg YG) on fecal microbiota. Boxplots represent the 5th percentile, 95th percentile, interquartile range (25–75%), and median. The black circle and error bars on the side of the boxplots represent the average value and SEM, respectively. *n* = 17 in the Bifal + Arg YG group and *n* = 16 in the placebo group. One subject in the Bifal + Arg YG group did not submit feces; therefore, it was regarded as a missing value. (**a**) Relative abundance of *Citrobacter*, *Escherichia*/*Shigella*, and *Enterococcus* in feces. * *p* < 0.05, Mann–Whitney *U* test. (**b**) Bacteroidetes:Firmicutes ratio in feces. * *p* < 0.05, Wilcoxon’s signed rank test. For the nonparametric data, paired *t*-test using log-transformed values was also performed; the differences were confirmed to be significant (* *p* < 0.05). (**c**) Number of *B. animalis* subsp. *lactis* in feces at week 12. *B. animalis* subsp. *lactis* was not detected in the feces of the placebo group (detection limit: 3.0 × 10^5^ cells/g of feces). (**d**) Principal component analysis of the fecal microbiota at week 12 (genus level). (**e**) Diversity analysis (chao 1) of fecal microbiota.

The results of our 16S rRNA gene amplicon sequence analysis are shown in Supplementary Tables S3–S7. To investigate whether intestinal microbiota is likely to produce putrescine, we focused on the relative abundance ratio of bacteria involved in intestinal putrescine production [16]. The relative abundance ratio of *Citrobacter*, representing bacteria possessing an arginine-dependent acid resistance system, was significantly higher in the Bifal + Arg YG group than that in the placebo group (*p* < 0.05 by Mann–Whitney *U* test; Figure 5a). Moreover, the relative abundance ratios of *Escherichia*/*Shigella*, representing bacteria possessing an arginine-dependent acid resistance system, and *Enterococcus*, representing bacteria possessing an agmatine deiminase system, were 5 times and 16 times higher in the Bifal + Arg YG group than those in the placebo group, respectively (Figure 5a). At week 12, the Bifal + Arg YG group showed a significant decrease in the relative abundance of Bacteroidetes (*p* < 0.05 by Wilcoxon’s signed rank test; Supplementary Table S2). In addition, the Bifal + Arg YG group showed a significant decrease in the Bacteroidetes/Firmicutes ratio (*p* < 0.05 by Wilcoxon’s signed rank test; Figure 5b). While the fecal bacterial count for *B. animalis* subsp. *lactis* in the Bifal + Arg group was (3.4 ± 0.7) × 10^9^ cells/g of feces, these bacteria were not detected in the placebo group (detection limit: 3.0 × 10^5^ cells/g of feces; Figure 5c). Principal component analysis and diversity analysis were subsequently performed to compare the fecal microbiota of each group at 0 and 12 weeks. Interestingly, no differences were observed between the two groups or within the groups at 0 and 12 weeks (Figure 5d,e, and Supplementary Figure S4).

### 3.8. Safety

Although one subject in the placebo group experienced diarrhea and withdrew from the clinical test, no other adverse events related to the yogurt treatment were observed for the subjects in either the Bifal + Arg YG or placebo group. Therefore, the tested yogurt is likely to be safe for consumption.

## 4. Discussion

We investigated the effect of consuming yogurt containing *B. animalis* subsp. *lactis* and Arg on endothelial function in healthy adults. Endothelial dysfunction increases the risk of developing cardiovascular disease [37]. Key RHI values <1.67 indicate endothelial dysfunction [22,23]. Here, the RHI increased from 1.50 to 1.81 after Bifal + Arg YG consumption, suggesting that Bifal + Arg YG intake restored endothelial function to normal levels. Moreover, blood pressure and cardiovascular disease are closely related, with systolic and diastolic blood pressure higher than 115 mmHg and 75 mmHg, respectively, being associated with an increased risk of disease development [38]. In the Bifal + Arg YG group, blood pressure decreased to near the baseline level, suggesting that the consumption of this yogurt improved blood pressure. Endothelial function and blood pressure strongly influence each other, and both deteriorate during atherosclerosis progression [39]. Therefore, simultaneous improvement in both parameters is not surprising and indicates that Bifal *+* Arg YG intake might help prevent the early process of atherosclerosis development. However, the observed changes in blood pressure could also be related to changes in smooth muscle cell reactivity and arterial compliance; further studies are needed to clarify this effect.

Colonic cells can absorb putrescine [40], which is utilized for spermidine and spermine biosynthesis [41]. Previously, we demonstrated that oral Arg administration in rats increased both fecal putrescine and blood spermidine concentration [15]. Moreover, various physiological activities of polyamines depend on the magnitude of their polarity [42]. Thus, although considerably substantial changes in spermidine and spermine levels induce autophagy, minor changes in putrescine levels do not [43,44]. Therefore, the improvement in endothelial function might be mediated by a putrescine-induced increase in serum spermidine biosynthesis. Blood spermidine-induced autophagy might be involved in both blood pressure reduction [5] and endothelial function improvement in mice [45], supporting our results. Thus, Bifal + Arg YG intake may induce microbial putrescine production, which is absorbed from the intestinal lumen and transported into the blood. Consequently, spermidine is biosynthesized, which promotes autophagy in endothelial cells, thereby improving endothelial function.

Other biomarkers further support the improvement of vascular endothelial function after Bifal + Arg YG consumption. Serum platelet [46], and triglyceride concentrations [47], two risk factors for atherosclerosis, decreased after Bifal + Arg YG consumption. Serum HDL-cholesterol, which is inversely correlated with cardiovascular disease incidence [48], increased significantly after Bifal + Arg YG consumption. These support the improvement of endothelial function by this yogurt.

Trimethylamine might be involved in the progression of atherosclerosis [49]. *B. animalis* subsp. *lactis* consumption has been shown to decrease the relative abundance of Lachnospiraceae and other Clostridiales, which might be involved in trimethylamine production [34]. However, in this study, the relative abundance of these bacterial groups and fecal trimethylamine concentration were unchanged. This might be attributed to the fecal collection method, which differed between the previous and current study. In addition, because dietary carnitine and choline influence microbial trimethylamine production [49], all subjects in the present study were provided identical meals on the day before feces collection. However, the subjects in the previous study ingested different meals. Therefore, further studies are required to clarify this discrepancy.

Arg is the substrate of NO synthase, which synthesizes the NO essential for blood pressure regulation and maintenance of endothelial function via vascular smooth muscle regulation [50]. A meta-analysis of clinical trials showed that oral administration of Arg lowers blood pressure effectively [51]. However, an Arg intake of 6 g/day does not affect blood pressure [52], and to our knowledge, no study has reported Arg intake below this level to be effective in improving blood pressure. The Bifal + Arg YG used in our study contained only 0.6 g Arg. This suggests that the improved endothelial function and decreased blood pressure after Bifal + Arg YG consumption was not caused by Arg-derived NO but might be modulated by the putrescine-induced increase in serum spermidine. Moreover, the serum NO_2_/NO_3_ concentration was unchanged after Bifal + Arg YG consumption, further supporting our conclusions.

According to the response to injury theory [53], the first stage of atherosclerosis is endothelial dysfunction, followed by the induction of an inflammatory response and atherosclerotic plaque formation. In this study, no differences were observed in serum TNF-α or IL-1β concentrations between the treated and placebo groups. The risk of cardiovascular disease was shown to increase when the blood TNF-α concentration was >6 pg/mL [54]. However, the subjects in the present study only had a maximum of 0.94 pg/mL, indicating the absence of a significant inflammatory environment. Therefore, the anti-inflammatory activity of polyamines [7,8] was probably not related to the improvement in endothelial function in this study.

Regarding fecal microbiota, the relative abundance of *Citrobacter* and *Escherichia/Shigella*, representing bacteria possessing an arginine-dependent acid resistance system (acid-tolerance system), and *Enterococcus*, representing bacteria possessing an agmatine deiminase system (energy production system), was higher in the Bifal + Arg YG group than in the placebo group. This indicated that Bifal + Arg YG intake might enhance the intestinal microbiota composition to be more suitable for putrescine production by acid-producing bacteria, including *B. animalis* subsp. *lactis* [16]. However, the reason for the observed reductions in Bacteroidetes and the Bacteroidetes/Firmicutes ratio in Bifal + Arg YG-treated subjects is unclear. Although a controversial association between obesity and decreased Bacteroidetes/Firmicutes ratios in the intestinal microbiota has been suggested [55], the BMI of these individuals did not change in the present study. Thus, this relationship needs to be clarified in future studies. There are several recent human studies on heart failure and alteration in intestinal microbiome [56]. Similar to our study, Luedde at al. [57] studied fecal microbiome, and analyzed data using 16S rRNA gene amplicon sequencing, and reported that patients with heart failure experienced significant decreases in levels of Coriobacteriaceae, Erysipelotrichaceae, and Ruminococcaceae at the family level, and decreases in levels of *Blautia*, *Collinsella*, unclassified Erysipelotrichaceae, and unclassified Ruminococcaceae at the genus level, as compared to healthy controls. Kamo et al. [58] found that patients with heart failure had significantly decreased levels of *Clostridium* and *Dorea* at the genus level as compared to healthy controls. Kummen et al. [59] reported significant increases in the levels of *Prevotella*, *Hungatella*, and *Succiniclasticum* and decreases in the levels of *Anaerostipes*, *Blautia*, *Coprococcus*, *Fusicatenibacter*, *Pseudobutyrivibrio*, *Faecalibacterium*, *Bifidobacterium*, *Eubacterium hallii* group, and an unknown genus in Lachnospiraceae at the genus level, in patients with heart failure as compared to healthy controls. However, in this study, the relative abundance of these bacterial groups was unchanged. Furthermore, although one of these studies found that bacterial diversity in patients with heart failure was decreased as compared to that in healthy controls, bacterial diversity was not changed during the experimental period. These analyses suggest that improvement of endothelial function triggered by this yogurt is independent of the intestinal bacterial composition.

The current study has some limitations. First, the subjects were all healthy Japanese individuals (BMI <30); we cannot be sure that the same results would be obtained if the subjects had severe atherosclerosis or were from other countries or ethnicities, with different dietary cultures. It is possible that the difference in the change in RHI was caused by the difference in the baseline RHI level between both groups. Therefore, the accurate effect needs to be confirmed by increasing the number of participants in future studies. Because there is a controversy regarding whether EndoPAT values are correlated with the results of FMD—a gold standard test of endothelial function—an additional study using FMD is indispensable in subsequent studies. Moreover, further studies are required to compare the administration of *B. animalis* subsp. *lactis* or Arg alone and together to confirm whether this strategy depends on the hybrid putrescine biosynthesis system. We do not know the reason why abdominal circumference in both groups was increased. It is estimated that this is caused by measurer’s bias because this value of all subjects unnaturally and uniformly increased more than 2 cm, and the measurer at week 12 differed from the one at week 0. Finally, as endo-peripheral arterial autophagy could not be analyzed, the possibility that other functions of spermidine led to this effect cannot be excluded.

Multiple clinical studies have reported the efficacy of probiotics in improving the blood lipid profile [60]. However, to our knowledge, direct improvement of endothelial function by probiotics has not yet been reported. Although one clinical test using *Lactobacillus casei* Shirota was conducted to assess the improvement of endothelial function, its efficacy was not observed [61]. Therefore, Bifal + Arg YG is the first functional food to demonstrate efficacy in improving endothelial function during the early process of atherosclerosis development in humans. Controlling intestinal bacterial metabolites might be a novel strategy to maintain host health. While it is challenging to control bacterial metabolites because of vast individual differences in human microbiomes and diets, our yogurt supplementation was effective in controlling target metabolites derived from intestinal microbiota and in enhancing endothelial function via altered physiological activity (autophagy). This study provides insights into atherosclerosis development and highlights a novel treatment option for at-risk patients.

## 5. Conclusions

Consuming yogurt containing *B. animalis* subsp. *lactis* and Arg may prevent or reduce the risk of atherosclerosis by upregulating blood spermidine levels, which subsequently induces autophagy. This is an innovative approach for the production of a desired bioactive metabolite derived from the gut microbiome to enhance endothelial function in humans.

## Figures and Tables

**Table 1:** Characteristics of subjects’ background and effects of yogurt containing *B. animalis* subsp. *lactis* and arginine on physical parameters, RHI, and blood pressure.

	Bifal + Arg Mean (SEM)			Placebo Mean (SEM)		
	Week 0	Week 12	Change	Week 0	Week 12	Change
Height, cm	163.7 (2.1)	163.9 (2.0)	+0.1 (0.1)	162.3 (2.2)	162.3 (2.2)	+0.1 (0.2)
Body weight, kg	64.0 (2.5)	64.5 (2.5)	+0.5 (0.3)	63.7 (2.6)	64.6 (2.6)	+0.9 (0.6)
Abdominal circumference, cm	79.4 (2.3)	82.8 (2.3) ^††^	+3.4 (0.6)	80.2 (2.2)	84.3 (1.8) ^††^	+4.1 (0.9)
Heart rate, bpm	72.9 (1.7)	70.9 (1.9)	−2.0 (2.0) ^#^	69.7 (1.5)	73.2 (2.4)	+3.5 (2.5) ^#^
BMI	23.8 (0.7)	23.9 (0.7)	+0.2 (0.1)	24.1 (0.6)	24.4 (0.7)	+0.3 (0.2)
RHI	1.50 (0.07)	1.81 (0.11) ^†^	+0.31 (0.12) ^#^	1.68 (0.11)	1.63 (0.12)	−0.07(0.18) ^#^
Systolic blood pressure, mmHg	122.4 (2.3)	120.2 (2.8)	−2.2 (2.3)	123.1 (3.4)	125.3 (2.5)	+2.1 (2.7)
Diastolic blood pressure, mmHg	77.9 (1.5)	75.1 (2.2)	−2.9 (2.1)	81.2 (2.2)	78.9 (1.9)	−2.25 (1.5)

All data are represented as mean ± SEM. Means are averages of all obtained data; relative changes are calculated from data, excluding outliers. Intra-group comparisons at week 0 and at week 12 were tested using the paired *t*-test (^†^ *p* < 0.05, ^††^ *p* < 0.01). Comparisons of change value between the Bifal + Arg YG and placebo groups were tested by two-way ANOVA (^#^ *p* < 0.05). All baseline × change interactions are shown in Supplementary Table S2. All *p*-values of difference are shown in Supplementary Table S1.

**Table 2:** Effects of yogurt containing *B. animalis* subsp. *lactis* and arginine on blood parameters.

	Bifal + Arg Mean (SEM)			Placebo Mean (SEM)		
	Week 0	Week 12	Change	Week 0	Week 12	Change
White blood cell count, μL	6315 (316)	5945 (326)	−370 (307)	5641 (347)	5352 (240)	−123 (395)
Erythrocyte count, 10^4^/μL	482.7(11.7)	478.6 (10.9)	−4.1 (5.1)	479.6(12.1)	470.5 (11.5) ^†^	−9.1 (3.2)
Hemoglobin, g/dL	14.0 (0.44)	14.0 (0.4)	−0.01 (0.24)	13.9 (0.4)	13.6 (0.38) ^†^	−0.32 (0.12)
Hematocrit, %	43.5 (1.1)	43.4 (1.1)	−0.04 (0.5)	42.8 (0.9)	42.4 (0.98)	−0.44 (0.34)
Platelet count, 10^4^/μL	27.9 (1.4)	25.6 (1.1) *^,††^	−2.2 (0.6) ^#^	27.7 (1.0)	28.4 (1.0) *	+0.7 (0.5) ^#^
Blood glucose level. mg/dL	89.17(2.92)	87.6 (2.2)	−1.56 (2.24)	92.31 (3.5)	91.13 (3.54)	+0.07 (2.59)
Total cholesterol, mg/dL	197.2 (6.6) *	196.3(6.0) **	−0.9 (4.4	223.8 (9.1) *	223.1(7.8) **	−0.7 (5.3)
Triglyceride, mg/dL	129.3(15.0)	103.9(12.9) ^†^	−25.4 (10.5)	104.8 (15.7)	125.1 (22.3)	+0.6 (8.0)
HDL-cholesterol, mg/dL	57.4 (3.3)	60.4 (3.4) ^†^	+3.0 (1.2)	54.4 (3.1)	55.6 (3.1)	+1.21 (1.8)
LDL-cholesterol, mg/dL	121.7 (6.9)	119.7 (6.3)	−2.0 (3.5)	129.2 (10.5)	131.1 (9.3)	+1.9 (4.3)
NO_2_/NO_3_, mM	N.T.	21.3 (1.5)	N.T.	N.T.	24.5 (2.1)	N.T
TNF-a, mM	N.T.	0.552 (0.03)	N.T.	N.T.	0.617 (0.05)	N.T.
IL-1, mM	N.T.	0.046 (0.01)	N.T.	N.T.	0.039 (0.01)	N.T.

All data are represented as mean ± SEM. Means are average of all obtained data; relative changes are calculated from data excluding outliers. Comparisons at week 0 and week 12 between the Bifal + Arg YG and placebo groups were tested by Student’s *t*-test (* *p* < 0.05, ** *p* < 0.01). Intra-group comparisons at week 0 and at week 12 were tested using the paired *t*-test (^†^ *p* < 0.05, ^††^ *p* < 0.01). Comparisons of change value between the Bifal + Arg YG and placebo groups were tested by two-way ANOVA (^#^ *p* < 0.05). All baseline × change interactions are shown in Supplementary Table S2. N.T., not test. All *p*-values of difference are shown in Supplementary Table S1.

## Supplementary Materials

The following are available online at https://www.mdpi.com/2072-6643/11/5/1188/s1, Figure S1: Menu items and nutritional value of the prepared diet, Figure S2: Accuracy of the GC-MS polyamine measurements, Figure S3: Exclusion of outliers with the Smirnov–Grubbs test, Figure S4: Principal component analysis of fecal microbiota at the phylum level, class level, order level, and family level, Table S1: Characteristics of subjects’ background and effects of yogurt containing *B. animalis* subsp. *lactis* and arginine on physical and blood parameters, Table S2: Baseline × change interactions by two-way ANOVA in comparison of change values between the Bifal + Arg YG and placebo groups, Table S3: Effect of consumption of yogurt containing *B. animalis* subsp. *lactis* and Arg (Bifal + Arg YG) on fecal microbiota composition (phylum level), Table S4: Effect of consumption of yogurt containing *B. animalis* subsp. *lactis* and Arg (Bifal + Arg YG) on fecal microbiota composition (class level), Table S5: Effect of consumption of yogurt containing *B. animalis* subsp. *lactis* and Arg (Bifal + Arg YG) on fecal microbiota composition (order level), Table S6: Effect of consumption of yogurt containing *B. animalis* subsp. *lactis* and Arg (Bifal + Arg YG) on fecal microbiota composition (family level), Table S7: Effect of consumption of yogurt containing *B. animalis* subsp. *lactis* and Arg (Bifal + Arg YG) on fecal microbiota composition (genus level).
