# Supplementary material for: Endothelial Function is improved by Inducing Microbial Polyamine Production in the Gut: A Randomized Placebo-Controlled Trial
Source: Nutrients. 2019 May 27;11(5):1188. doi: 10.3390/nu11051188 (PMC6566626; doi:10.3390/nu11051188)
Supplement: Supplementary file 1 [file nutrients-11-01188-s001.pdf]

Supplemental Materials

Endothelial Function is improved by Inducing Microbial Polyamine Production in the Gut: A Randomized Placebo-Controlled Trial

Mitsuharu Matsumoto [1,*], Yusuke Kitada [1], and Yuji Naito [2]

[1] Dairy Science and Technology Institute, Kyodo Milk Industry Co. Ltd., Tokyo 190-0182, Japan; y-kitada@meito.co.jp

[2] Molecular Gastroenterology and Hepatology, Kyoto Prefectural University of Medicine, Kyoto 602-8566, Japan; ynaito@koto.kpu-m.ac.jp

* Correspondence: m-matumoto@meito.co.jp; Tel.:+81-42-597-5911

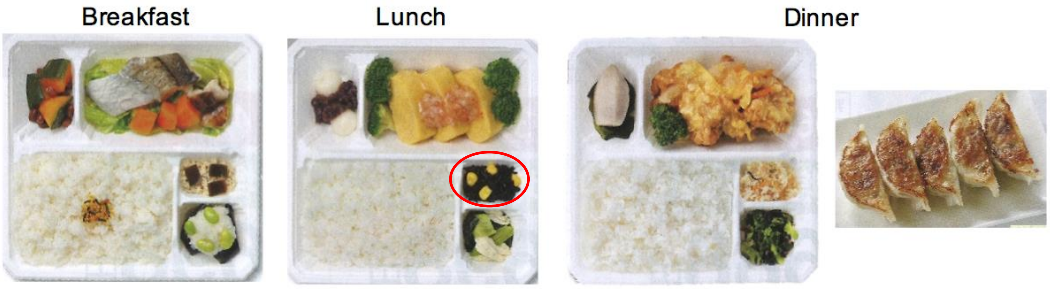

	Main menu	Calorie(kcal)	Protein(g)	Lipid(g)	Carbohydrate(g)	Dietary fiber(g)	Na(mg)	Total calories
Breakfast	1. Rice<br>2. Cod and chinese cabbage with japanese style runny sauce<br>3. Braised pumpkin<br>4. Deep-fried eggplant in Japanese broth	601	17.7	16.2	92.1	3.8	435	
Lunch	1. Rice<br>2. Japanese omelette with crab sauce<br>3. Simmered Hijiki seaweed<br>4. Sticky rice flour dumplings	606	16.7	13.4	100.5	2.9	521	
Dinner	1. Rice<br>2. Fried chicken with egg soup<br>3. Boiled spinach<br>4. Simmered taro<br>5. Panned dumpling	763	20.2	18.6	120.4	6.4	949	1970 kcal

Supplementary Figure S1. Menu items and nutritional value of the prepared diet. Hijiki, the marker for stool collection, is circled in red.

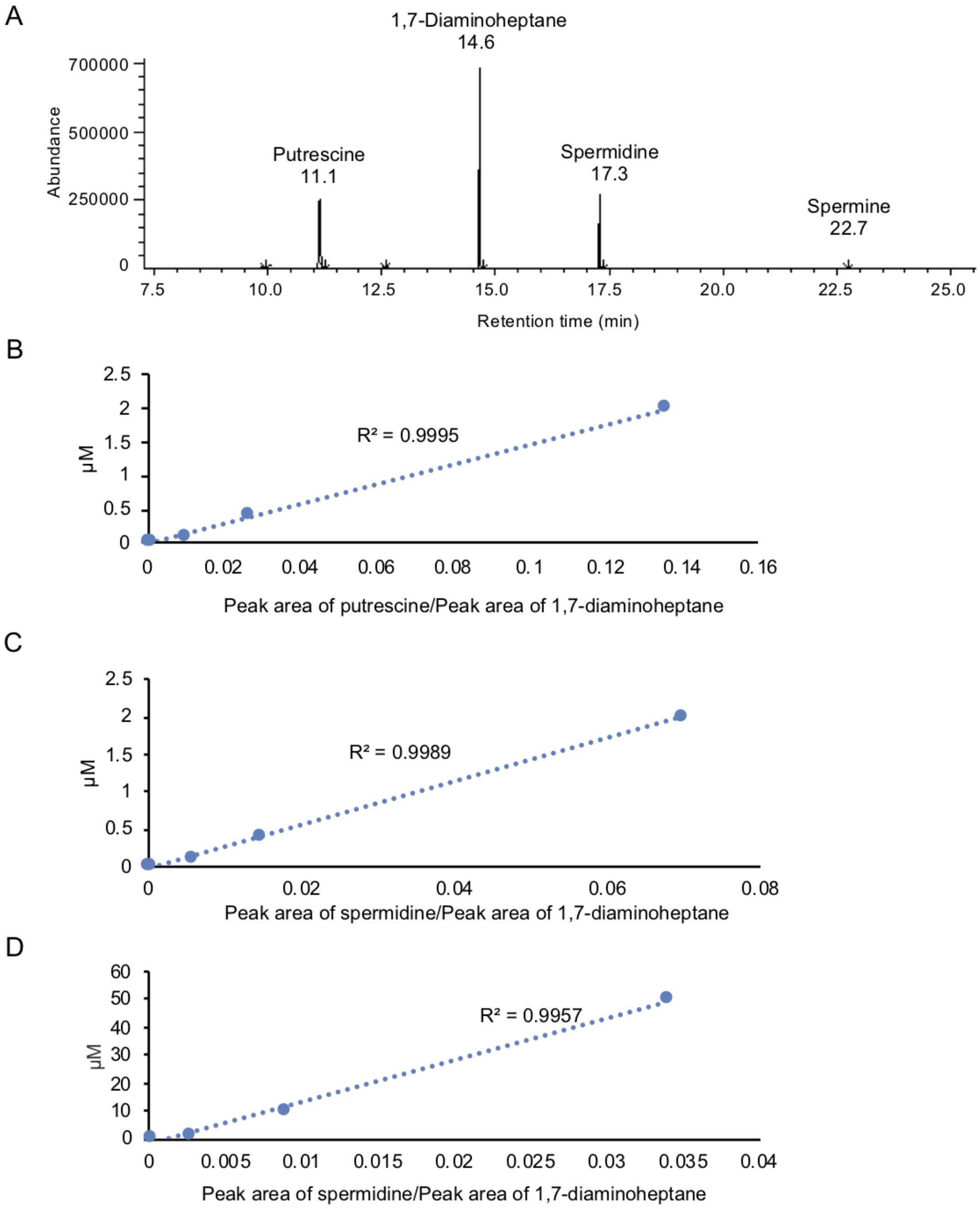

Supplementary Figure S2. Accuracy of the GC-MS polyamine measurements. (A) Chromatogram and retention time of putrescine, 1,4-diaminoheptane, and spermidine. (B) Calibration curve of putrescine. (C) Calibration curve of spermidine. (D) Calibration curve of spermine. The retention times of putrescine, spermidine, spermine and 1,4-diaminoheptane were 11.1 min, 17.3 min, 22.7 min and 14.6 min, respectively. Calibration curves were prepared by correcting the area of the standards with the area of 1,4-diaminoheptane as an internal standard. The correlation coefficients (R^2) of putrescine, spermidine, and spermine calibration curves were 0.9995, 0.9989 and 0.9957, respectively.

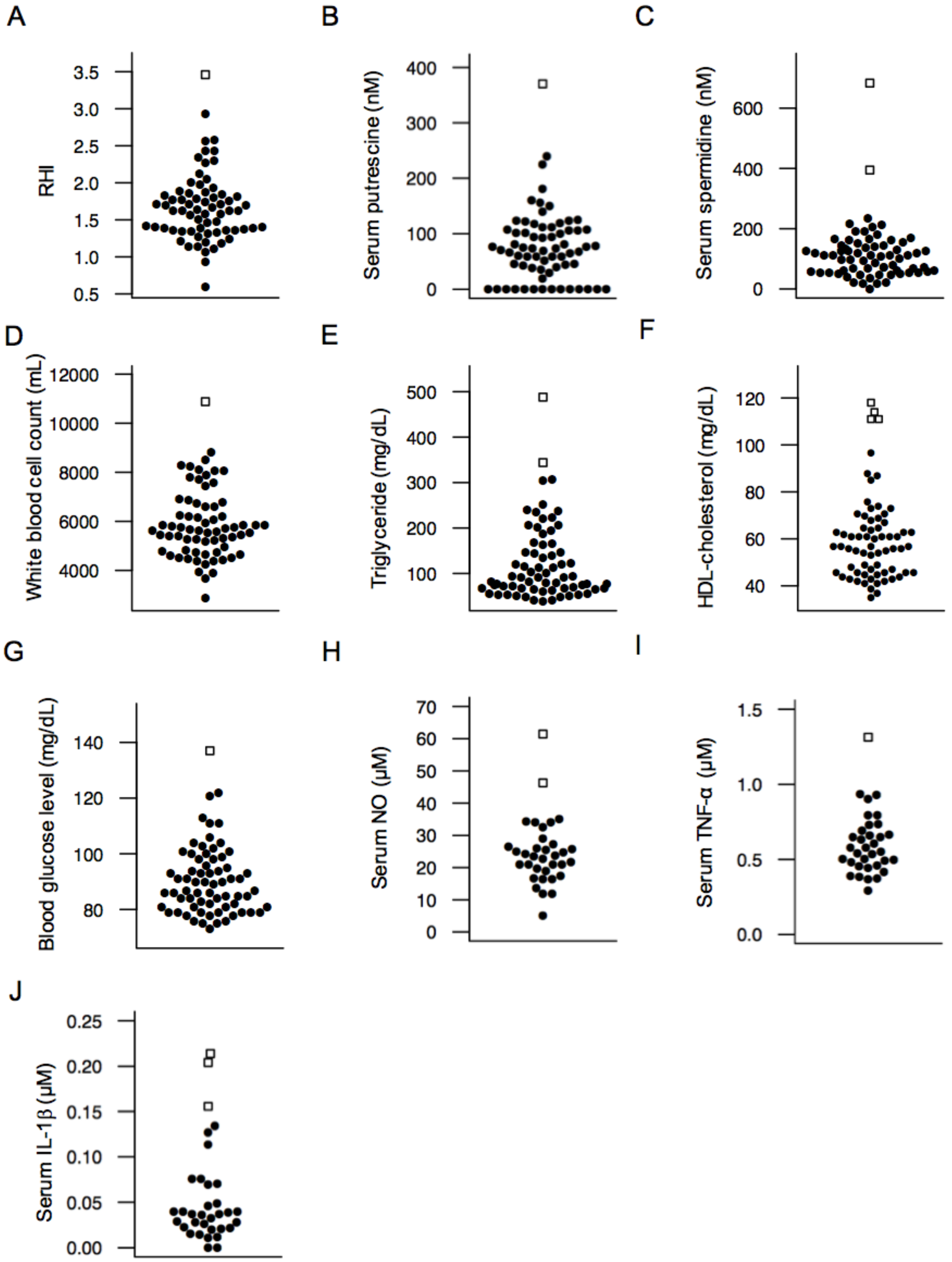

Supplementary Figure S3. Exclusion of outliers with the Smirnov–Grubbs test. The Smirnov-Grubbs test was performed on all subject data for each analysis: (A) RHI; (B) serum putrescine; (C) serum spermidine; (D) white blood cell count; (E) serum triglyceride; (F) serum high-density lipoprotein (HDL)-cholesterol; (G) blood glucose levels; (H) serum NO_2/NO_3; (I) serum TNF-◎; and (J) serum IL-1◎. Serum NO, serum TNF-◎; and serum IL-1◎ were not analyzed at week 0. The outlier data are indicated by open squares.

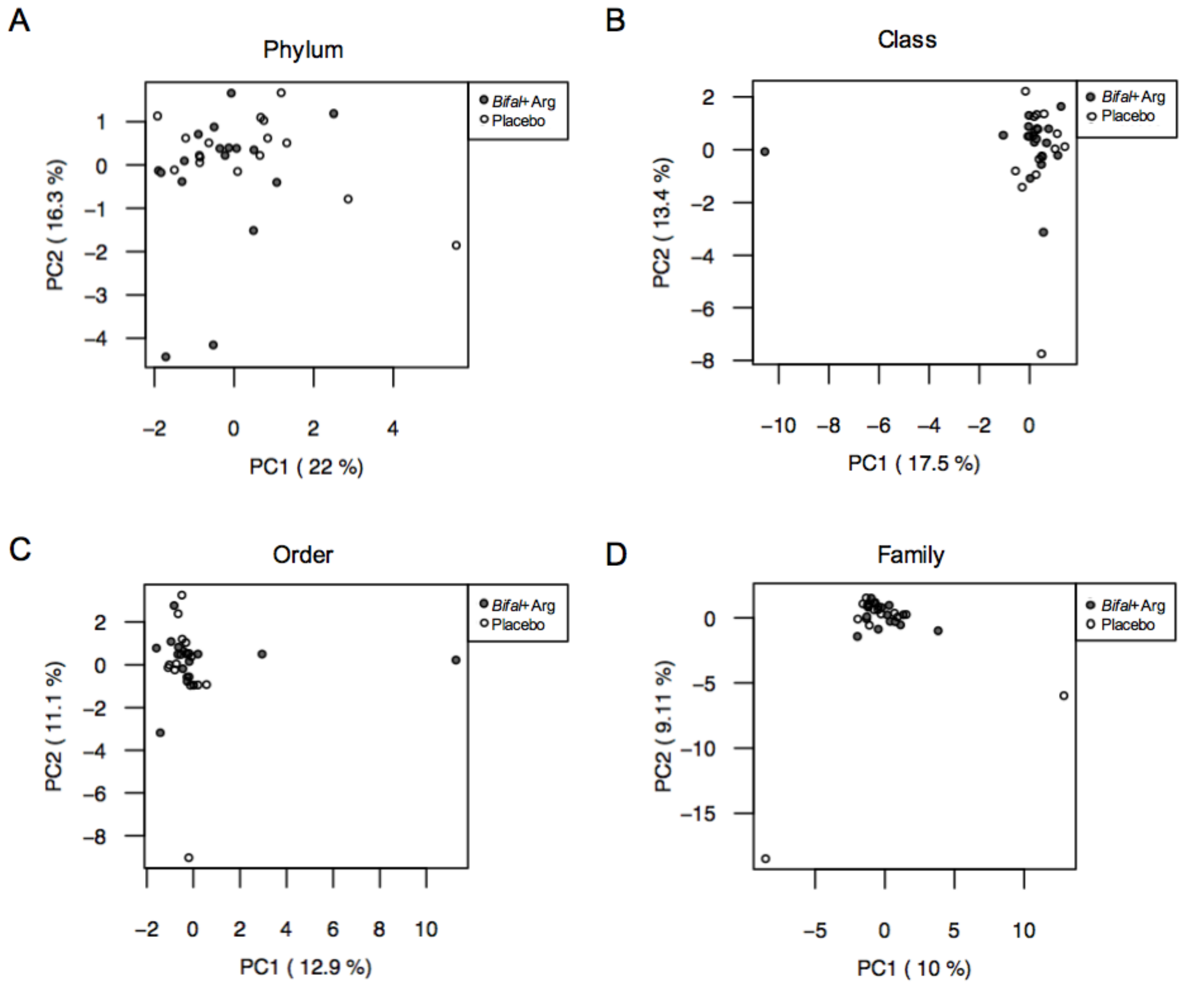

Supplementary Figure S4. Principal component analysis of fecal microbiota at the phylum level (A), class level (B), order level (C), and family level (D).

Supplementary Table 1. Characteristics of subjects' background and effects of yogurt containing *B. animalis* subsp. *lactis* and arginine on physical and blood parameters.

	Bifal+Arg Mean (SEM)			Placebo			Difference (*p*-value)				
							Bifal+Arg vs. Placebo			Week 0 vs. Week 12	
	Week 0	Week 12	Change	Week 0	Week 12	Change	Week 0	Week 12	Change	*Bifal*+Arg	Placebo
Physical examination											
Height, cm	163.7 (2.1)	163.9 (2.0)	+0.1 (0.1)	162.3 (2.2)	162.3 (2.2)	+0.1 (0.2)	0.636	0.308	0.486	0.288	0.718
Body weight, kg	64.0 (2.5)	64.5 (2.5)	+0.5 (0.3)	63.7 (2.6)	64.6 (2.6)	+0.9 (0.6)	0.936	0.496	0.450	0.083	0.142
Abdominal circumference, cm	79.4 (2.3)	82.8 (2.3)	+3.4 (0.6)	80.2 (2.2)	84.3 (1.8)	+4.1 (0.9)	0.798	0.307	0.386	0.001*	0.001*
Heart rate, bpm	72.9 (1.7)	70.9 (1.9)	-2.0 (2.0)	69.7 (1.5)	73.2 (2.4)	+3.5 (2.5)	0.158	0.230	0.025*	0.328	0.185
BMI	23.8 (0.7)	23.9 (0.7)	+0.2 (0.1)	24.1 (0.6)	24.4 (0.7)	+0.3 (0.2)	0.762	0.323	0.405	0.198	0.142
Endothelial function/blood pressure											
RHI	1.50 (0.07)	1.81 (0.11)	+0.31 (0.12)	1.68 (0.11)	1.63 (0.12)	-0.07(0.18)	0.174	0.132	0.017*	0.010*	0.356
Systolic blood pressure, mmHg	122.4 (2.3)	120.2 (2.8)	-2.2 (2.3)	123.1 (3.4)	125.3 (2.5)	+2.13 (2.7)	0.855	0.097	0.131	0.184	0.223
Diastolic blood pressure, mmHg	77.9 (1.5)	75.1 (2.2)	-2.9 (2.1)	81.2 (2.2)	78.9 (1.9)	-2.25 (1.5)	0.229	0.097	0.405	0.093	0.073
Blood biochemical analyses											
White blood cell count , mL	6315 (316)	5945 (326)	-370 (307)	5641 (347)	5352 (240)	-123 (395)	0.160	0.086	0.303	0.245	0.759
Erythrocyte count, 10^4/mL	482.7(11.7)	478.6(10.9)	-4.1 (5.1)	479.6(12.1)	470.5 (11.5)	-9.1 (3.2)	0.853	0.306	0.349	0.434	0.013*
Hemoglobin, g/dL	14.03(0.44)	140.2 (0.4)	-0.01 (0.24)	13.92 (0.4)	13.6 (0.38)	-0.32 (0.12)	0.848	0.226	0.255	0.964	0.022*
Hematocrit, %	43.5 (1.1)	43.4 (1.1)	-0.04 (0.5)	42.8 (0.9)	42.39 (0.98)	-0.44 (0.34)	0.655	0.238	0.364	0.932	0.221
Platelet count, 10^4/mL	27.9 (1.4)	25.6 (1.1)	-2.2 (0.6)	27.7 (1.0)	28.4 (1.0)	+0.7 (0.5)	0.938	0.040*	0.017*	0.003*	0.170
Blood glucose level. mg/dL	89.17(2.92)	87.6 (2.2)	-1.56 (2.24)	92.31 (3.5)	91.13 (3.54)	+0.07(2.59)	0.492	0.137	0.346	0.497	0.980
Total cholesterol, mg/dL	197.2 (6.6)	196.3 (6.0)	-0.9 (4.4	223.8 (9.1)	223.1 (7.8)	-0.7 (5.3)	0.022*	0.006	0.491	0.829	0.898
Triglyceride, mg/dL	129.3(15.0)	103.9(12.9)	-25.4 (10.5)	104.8(15.7)	125.1 (22.3)	0.6 (8.0)	0.285	0.202	0.085	0.027*	0.944
HDL-cholesterol, mg/dL	57.4 (3.3)	60.4 (3.4)	3.0 (1.2)	54.4 (3.1)	55.6 (3.1)	+1.21 (1.8)	0.536	0.134	0.313	0.019*	0.502
LDL-cholesterol, mg/dL	121.7 (6.9)	119.7 (6.3)	-2.0 (3.5)	129.2(10.5)	131.1 (9.3)	+1.9 (4.3)	0.547	0.154	0.341	0.572	0.661
NO2/NO3, mM	N.T.	21.3 (1.5)	N.T.	N.T.	24.5 (2.1)	N.T	-	0.103	-	-	-
Inflammatory markers											
TNF-a, mM	N.T.	0.552(0.03)	N.T.	N.T.	0.617 (0.05)	N.T.	-	0.137	-	-	-
IL-1, mM	N.T.	0.046(0.01)	N.T.	N.T.	0.039 (0.01)	N.T.	-	0.294	-	-	-
Urinalysis											
Specific gravity	1.015 (0.002)	1.017 (0.002)	+0.002 (0.002)	1.015 (0.002)	1.017 (0.002)	+0.002 (0.002)	0.888	0.499	0.445	0.257	0.501
pH	6.3 (0.1)	6.1 (0.2)	-0.1 (0.2)	6.2 (0.2)	6.0 (0.2)	-0.2 (0.2)	0.769	0.268	0.378	0.451	0.371

All data are represented as mean ± SEM. Means are average of all obtained data; change values are calculated from data excluding outliers. Comparisons at week 0 and week 12 between the *Bifal*+Arg YG and placebo groups were tested by Student's *t*-test. Intra-group comparisons at week 0 and at week 12 were tested using the paired *t*-test. Comparisons of change value between the *Bifal*+Arg YG and placebo groups were tested by two-way ANOVA. All baseline × change interactions are shown in Supplementary Table S2. N.T., not test.

Supplementary Table S2. Baseline × change interactions by two-way ANOVA in comparison of change values between the *Bifal*+Arg YG and placebo groups.

	baseline × change interaction	Difference (*p* -value)
Physical examination		
Height, cm	0.653	0.486
Body w eight, kg	0.866	0.450
Abdominal circumference, cm	0.970	0.386
Heart rate, bpm	0.028*	0.025*
BMI	0.900	0.405
Endothelial function and blood pressure		
RHI	0.023	0.017*
Systolic blood pressure, mmHg	0.509	0.131
Diastolic blood pressure, mmHg	0.487	0.405
Blood biochemical analyses		
White blood cell count , mL	0.111	0.303
Erythrocyte count, 10^4/mL	0.921	0.349
Hemoglobin, g/dL	0.770	0.255
Hematocrit, %	0.868	0.364
Platelet count, 10^4/mL	0.111	0.017*
Blood glucose level. mg/dL	0.790	0.346
Total cholesterol, mg/dL	0.044	0.491
Triglyceride, mg/dL	0.061	0.085
HDL-cholesterol, mg/dL	0.820	0.313
LDL-cholesterol, mg/dL	0.792	0.341
NO2/NO3, μM		-
Urinalysis		
Specific gravity	0.844	0.445
pH	0.962	0.378

*Significant difference

Supplementary Table S3. Effect of consumption of yogurt containing *B. animalis* subsp. *lactis* and Arg (*Bifal*+Arg YG) on fecal microbiota composition (phylum level)

	Bifal+Arg				Placebo				Difference (*p*-value)			
	Week 0		Week 12		Week 0		Week 12		*Bifal*+Arg VS. Placebo		Week 0 VS. Week 12	
Phylum	Mean	SEM	Mean	SEM	Mean	SEM	Mean	SEM	Week 0	Week 12	*Bifal*+Arg	Placebo
Other phylum	0.4468	0.1197	0.5738	0.2846	0.8376	0.5371	0.7690	0.3498	0.73	1.00	0.93	1.00
Actinobacteria	5.7570	1.5603	10.7320	2.7014	6.7687	1.2355	10.1301	2.1919	0.96	0.94	0.32	1.00
Bacteroidetes	23.7262	4.7709	6.2236	1.6113	15.3235	3.6120	11.0537	3.3907	0.73	0.81	0.02	1.00
Candidatus Saccharibacteria	0.0058	0.0016	0.0099	0.0026	0.0066	0.0020	0.0083	0.0035	0.73	1.00	0.78	1.00
Cyanobacteria/Chloroplast	0.0038	0.0019	0.0068	0.0037	0.0061	0.0034	0.0021	0.0012	0.73	1.00	1.00	1.00
Firmicutes	66.4289	4.9578	79.8957	3.1530	75.6912	3.1742	76.8261	4.0392	0.73	0.81	0.32	1.00
Fusobacteria	1.1084	0.8079	0.0560	0.0414	0.5837	0.3067	0.7442	0.6998	0.98	0.81	0.34	1.00
Lentisphaerae	0.0012	0.0012	0.0000	0.0000	0.0003	0.0003	0.0000	0.0000	-	1.00	1.000	1.00
Proteobacteria	2.4831	0.6833	2.4418	1.0007	0.7676	0.2449	0.3857	0.0764	0.73	0.37	0.93	1.00
Spirochaetes	0.0105	0.0105	0.0033	0.0033	0.0084	0.0080	0.0737	0.0645	0.73	1.00	1.00	1.00
Synergistetes	0.0004	0.0004	0.0050	0.0050	0.0002	0.0002	0.0007	0.0005	0.90	1.00	1.00	1.00
Verrucomicrobia	0.0280	0.0185	0.0521	0.0319	0.0062	0.0058	0.0065	0.0065	0.73	0.94	1.00	1.00

Comparisons between the *Bifal*+Arg YG and placebo groups were tested using Mann-Whitney *U* tests. Comparisons at week 0 and at week 12 were tested using Wilcoxon signed rank tests. *p*-values were adjusted using the Benjamini and Hochberg method [1], and adjusted *p*-values less than 0.05 are indicated by red circles.

References

1. Benjamini, Y.; Hochberg, Y. Controlling the false discovery rate: a practical and powerful approach to multiple testing. *J. R. Statist. Soc. B (Methodological)* **1995**, 289-300.

Supplementary Table S4. Effect of consumption of yogurt containing *B. animalis* subsp. *lactis* and Arg (*Bifal*+Arg YG) on fecal microbiota composition (class level)

Class	*Bifal*+Arg				Placebo				Difference (*p*-value)			
	Week 0		Week 12		Week 0		Week 12		*Bifal*+Arg VS. Placebo		Week 0 VS. Week 12	
	Mean	SEM	Mean	SEM	Mean	SEM	Mean	SEM	Week 0	Week 12	*Bifal*+Arg	Placebo
Other phylum, Other class	0.4468	0.1197	0.5738	0.2846	0.8376	0.5371	0.7690	0.3498	1.00	0.81	1.00	1.00
Actinobacteria, Actinobacteria	5.7570	1.5603	10.7320	2.7014	6.7687	1.2355	10.1301	2.1919	1.00	1.00	0.37	1.00
Bacteroidetes, Other class	0.0398	0.0188	0.0246	0.0139	0.0065	0.0052	0.0002	0.0002	1.00	0.64	1.00	0.79
Bacteroidetes, Bacteroidia	23.6864	4.7714	6.1990	1.6068	15.3170	3.6128	11.0534	3.3907	1.00	0.81	0.03	1.00
Candidatus Saccharibacteria, Other class	0.0058	0.0016	0.0099	0.0026	0.0066	0.0020	0.0083	0.0035	1.00	0.81	0.89	1.00
Cyanobacteria/Chloroplast, Chloroplast	0.0038	0.0019	0.0068	0.0037	0.0054	0.0034	0.0021	0.0012	1.00	0.81	1.00	1.00
Cyanobacteria/Chloroplast, Cyanobacteria	0.0000	0.0000	0.0000	0.0000	0.0006	0.0006	0.0000	0.0000	1.00	-	-	1.00
Firmicutes, Other class	0.1680	0.0650	0.1194	0.0336	0.2677	0.1759	0.2781	0.1460	1.00	1.00	1.00	1.00
Firmicutes, Bacilli	3.1051	0.9433	11.1388	3.3192	6.3936	3.8538	6.7904	1.7396	1.00	0.81	0.044	0.79
Firmicutes, Clostridia	55.2614	5.5620	63.7566	3.9941	59.2689	4.8674	60.6042	4.2600	1.00	0.86	0.89	1.00
Firmicutes, Erysipelotrichia	4.4262	1.3508	3.9570	0.7132	4.2730	1.1484	6.4483	1.5434	1.00	0.81	1.00	1.00
Firmicutes, Negativicutes	3.4682	1.9776	0.9240	0.4764	5.4880	2.2003	2.7051	0.9876	1.00	0.81	0.04	1.00
Fusobacteria, Fusobacteriia	1.1084	0.8079	0.0560	0.0414	0.5837	0.3067	0.7442	0.6998	1.00	1.00	0.48	1.00
Lentisphaerae, Lentisphaeria	0.0012	0.0012	0.0000	0.0000	0.0003	0.0003	0.0000	0.0000	1.00	-	1.00	1.00
Proteobacteria, Other class	0.0010	0.0010	0.0013	0.0013	0.0004	0.0004	0.0000	0.0000	1.00	1.00	1.00	1.00
Proteobacteria, Alphaproteobacteria	0.0006	0.0004	0.0043	0.0040	0.0000	0.0000	0.0007	0.0005	1.00	1.00	1.00	1.00
Proteobacteria, Betaproteobacteria	0.4481	0.2022	0.2814	0.1308	0.2436	0.1498	0.1060	0.0500	1.00	0.81	1.00	1.00
Proteobacteria, Deltaproteobacteria	0.0331	0.0129	0.0126	0.0081	0.0640	0.0322	0.0229	0.0134	1.00	1.00	0.48	1.00
Proteobacteria, Epsilonproteobacteria	0.0098	0.0098	0.0000	0.0000	0.0003	0.0003	0.0000	0.0000	1.00	-	1.00	1.00
Proteobacteria, Gammaproteobacteria	1.9904	0.6231	2.1421	1.0038	0.4593	0.1508	0.2561	0.0779	1.00	0.81	1.00	1.00
Spirochaetes, Spirochaetia	0.0105	0.0105	0.0033	0.0033	0.0084	0.0080	0.0737	0.0645	1.00	0.81	1.00	1.00
Synergistetes, Synergistia	0.0004	0.0004	0.0050	0.0050	0.0002	0.0002	0.0007	0.0005	1.00	1.00	1.00	1.00
Verrucomicrobia, Verrucomicrobiae	0.0280	0.0185	0.0521	0.0319	0.0062	0.0058	0.0065	0.0065	1.00	0.81	1.00	1.00

Comparisons between the *Bifal*+Arg YG and placebo groups were tested using Mann-Whitney *U* tests. Comparisons at week 0 and at week 12 were tested using Wilcoxon signed rank tests. *p*-values were adjusted using the Benjamini and Hochberg method [1], and adjusted *p*-values less than 0.05 are indicated by red circles.

References

1. Benjamini, Y.; Hochberg, Y. Controlling the false discovery rate: a practical and powerful approach to multiple testing. *J. R. Statist. Soc. B (Methodological)* **1995**, 289-300.

Supplementary Table S5. Effect of consumption of yogurt containing *B. animalis* subsp. *lactis* and Arg (*Bifal*+Arg YG) on fecal microbiota composition (order level)

Order	*Bifal*+Arg				Placebo				Difference (*p*-value)			
	Week 0		Week 12		Week 0		Week 12		*Bifal*+Arg VS. Placebo		Week 0 VS. Week 12	
	Mean	SEM	Mean	SEM	Mean	SEM	Mean	SEM	Week 0	Week 12	*Bifal*+Arg	Placebo
Other phylum, Other class, Other order	0.4468	0.1197	0.5738	0.2846	0.8376	0.5371	0.7690	0.3498	1.00	1.00	1.00	1.00
Actinobacteria, Actinobacteria, Other order	0.0000	0.0000	0.0000	0.0000	0.0000	0.0000	0.0003	0.0003	-	0.89	-	1.00
Actinobacteria, Actinobacteria, Acidimicrobiales	0.0006	0.0006	0.0000	0.0000	0.0000	0.0000	0.0000	0.0000	1.00	-	1.00	-
Actinobacteria, Actinobacteria, Actinomycetales	0.0205	0.0037	0.0175	0.0043	0.0392	0.0217	0.0146	0.0043	1.00	0.98	1.00	1.00
Actinobacteria, Actinobacteria, Bifidobacteriales	5.4790	1.5724	10.2765	2.6920	6.3311	1.2241	9.6698	2.1906	1.00	1.00	0.55	1.00
Actinobacteria, Actinobacteria, Coriobacteriales	0.2569	0.0629	0.4381	0.0649	0.3985	0.1183	0.4455	0.1057	1.00	0.92	0.15	1.00
Bacteroidetes, Other class, Other order	0.0398	0.0188	0.0246	0.0139	0.0065	0.0052	0.0002	0.0002	1.00	0.89	1.00	1.00
Bacteroidetes, Bacteroidia, Bacteroidales	23.6864	4.7714	6.1990	1.6068	15.3170	3.6128	11.0534	3.3907	1.00	0.89	0.05	1.00
Candidatus Saccharibacteria, Other class, Other order	0.0058	0.0016	0.0099	0.0026	0.0066	0.0020	0.0083	0.0035	1.00	0.89	1.00	1.00
Cyanobacteria/Chloroplast, Chloroplast, Other order	0.0038	0.0019	0.0068	0.0037	0.0054	0.0034	0.0021	0.0012	1.00	0.89	1.00	1.00
Cyanobacteria/Chloroplast, Cyanobacteria, Other order	0.0000	0.0000	0.0000	0.0000	0.0006	0.0006	0.0000	0.0000	-	1.00	-	1.00
Firmicutes, Other class, Other order	0.1680	0.0650	0.1194	0.0336	0.2677	0.1759	0.2781	0.1460	1.00	1.00	1.00	1.00
Firmicutes, Bacilli, Other order	0.0000	0.0000	0.0006	0.0006	0.0000	0.0000	0.0004	0.0004	1.00	0.92	1.00	1.00
Firmicutes, Bacilli, Bacillales	0.0340	0.0080	0.0742	0.0420	0.1681	0.1404	0.0455	0.0162	1.00	0.89	1.000	1.00
Firmicutes, Bacilli, Lactobacillales	3.0711	0.9432	11.0639	3.3219	6.2255	3.8288	6.7445	1.7435	1.00	1.00	0.07	1.00
Firmicutes, Clostridia, Other order	0.0083	0.0065	0.0028	0.0028	0.0188	0.0166	0.0186	0.0177	1.00	0.89	1.00	1.00
Firmicutes, Clostridia, Clostridiales	55.2531	5.5594	63.7538	3.9934	59.2501	4.8702	60.5856	4.2633	1.00	0.89	1.00	1.00
Firmicutes, Erysipelotrichia, Erysipelotrichales	4.4262	1.3508	3.9570	0.7132	4.2730	1.1484	6.4483	1.5434	1.00	0.89	1.00	1.00
Firmicutes, Negativicutes, Selenomonadales	3.4682	1.9776	0.9240	0.4764	5.4880	2.2003	2.7051	0.9876	1.00	1.00	0.07	1.00
Fusobacteria, Fusobacteriia, Fusobacteriales	1.1084	0.8079	0.0560	0.0414	0.5837	0.3067	0.7442	0.6998	1.00	0.89	0.55	1.00
Lentisphaerae, Lentisphaeria, Victivallales	0.0012	0.0012	0.0000	0.0000	0.0003	0.0003	0.0000	0.0000	1.00	0.89	1.00	1.00
Proteobacteria, Other class, Other order	0.0010	0.0010	0.0013	0.0013	0.0004	0.0004	0.0000	0.0000	1.00	0.89	1.00	1.00
Proteobacteria, Alphaproteobacteria, Caulobacterales	0.0003	0.0003	0.0000	0.0000	0.0000	0.0000	0.0004	0.0004	1.00	0.89	1.00	1.00
Proteobacteria, Alphaproteobacteria, Other order	0.0003	0.0003	0.0040	0.0040	0.0000	0.0000	0.0000	0.0000	1.00	-	1.00	-
Proteobacteria, Alphaproteobacteria, Rhizobiales	0.0000	0.0000	0.0000	0.0000	0.0000	0.0000	0.0003	0.0003	-	1.00	-	1.00
Proteobacteria, Alphaproteobacteria, Sphingomonadales	0.0000	0.0000	0.0003	0.0003	0.0000	0.0000	0.0000	0.0000	1.00	-	1.00	-
Proteobacteria, Betaproteobacteria, Other order	0.0119	0.0090	0.0003	0.0003	0.0386	0.0373	0.0029	0.0022	1.00	0.89	0.55	1.00
Proteobacteria, Betaproteobacteria, Burkholderiales	0.4363	0.1995	0.2812	0.1306	0.2049	0.1483	0.1031	0.0503	1.00	0.89	1.00	1.00
Proteobacteria, Deltaproteobacteria, Desulfovibrionales	0.0331	0.0129	0.0126	0.0081	0.0640	0.0322	0.0229	0.0134	1.00	1.00	0.56	1.00
Proteobacteria, Epsilonproteobacteria, Campylobacterales	0.0098	0.0098	0.0000	0.0000	0.0003	0.0003	0.0000	0.0000	1.00	0.89	1.00	1.00
Proteobacteria, Gammaproteobacteria, Enterobacteriales	1.9746	0.6232	2.1152	0.9995	0.3964	0.1513	0.2160	0.0773	0.73	1.00	1.00	1.00
Proteobacteria, Gammaproteobacteria, Other order	0.0000	0.0000	0.0003	0.0003	0.0000	0.0000	0.0000	0.0000	1.00	-	1.00	-
Proteobacteria, Gammaproteobacteria, Pasteurellales	0.0156	0.0062	0.0267	0.0119	0.0627	0.0289	0.0399	0.0250	1.00	1.00	1.00	1.00
Proteobacteria, Gammaproteobacteria, Pseudomonadales	0.0002	0.0002	0.0000	0.0000	0.0002	0.0002	0.0000	0.0000	1.00	0.89	1.00	1.00
Proteobacteria, Gammaproteobacteria, Xanthomonadales	0.0000	0.0000	0.0000	0.0000	0.0000	0.0000	0.0003	0.0003	-	1.00	-	1.00
Spirochaetes, Spirochaetia, Spirochaetales	0.0105	0.0105	0.0033	0.0033	0.0084	0.0080	0.0737	0.0645	1.00	1.00	1.00	1.00
Synergistetes, Synergistia, Synergistales	0.0004	0.0004	0.0050	0.0050	0.0002	0.0002	0.0007	0.0005	1.00	0.89	1.00	1.00
Verrucomicrobia, Verrucomicrobiae, Verrucomicrobiales	0.0280	0.0185	0.0521	0.0319	0.0062	0.0058	0.0065	0.0065	1.00	1.00	1.00	1.00

Comparisons between the *Bifal*+Arg YG and placebo groups were tested using Mann-Whitney *U* tests. Comparisons at week 0 and at week 12 were tested using Wilcoxon signed rank tests. *p*-values were adjusted using the Benjamini and Hochberg method [1].

References

1. Benjamini, Y.; Hochberg, Y. Controlling the false discovery rate: a practical and powerful approach to multiple testing. *J. R. Statist. Soc. B (Methodological)* **1995**, 289-300.

Supplementary Table S6. Effect of consumption of yogurt containing *B. animalis* subsp. *lactis* and Arg (*Bifal*+Arg YG) on fecal microbiota composition (family level)

	Bifal+Arg				Placebo				Difference (*p*-value)			
	Week 0		Week 12		Week 0		Week 12		*Bifal*+Arg VS. Placebo		Week 0 VS. Week 12	
Family	Mean	SEM	Mean	SEM	Mean	SEM	Mean	SEM	Week 0	Week 12	*Bifal*+Arg	Placebo
Other phylum, Other class, Other order, Other family	0.4468	0.1197	0.5738	0.2846	0.8376	0.5371	0.7690	0.3498	1.00	0.87	1.00	1.00
Actinobacteria, Actinobacteria, Other order, Other family	0.0000	0.0000	0.0000	0.0000	0.0000	0.0000	0.0003	0.0003	-	0.87	-	1.00
Actinobacteria, Actinobacteria, Acidimicrobiales, Other family	0.0006	0.0006	0.0000	0.0000	0.0000	0.0000	0.0000	0.0000	1.00	-	1.00	1.00
Actinobacteria, Actinobacteria, Actinomycetales, Actinomycetaceae	0.0141	0.0020	0.0115	0.0031	0.0274	0.0146	0.0076	0.0030	1.00	0.87	1.00	1.00
Actinobacteria, Actinobacteria, Actinomycetales, Other family	0.0014	0.0008	0.0003	0.0003	0.0014	0.0006	0.0013	0.0006	1.00	0.87	1.00	1.00
Actinobacteria, Actinobacteria, Actinomycetales, Corynebacteriaceae	0.0018	0.0012	0.0017	0.0010	0.0003	0.0003	0.0000	0.0000	1.00	0.87	1.00	1.00
Actinobacteria, Actinobacteria, Actinomycetales, Dermabacteraceae	0.0000	0.0000	0.0004	0.0004	0.0000	0.0000	0.0000	0.0000	-	1.00	1.00	-
Actinobacteria, Actinobacteria, Actinomycetales, Micrococcaceae	0.0033	0.0012	0.0030	0.0015	0.0099	0.0074	0.0056	0.0022	1.00	0.87	1.00	1.00
Actinobacteria, Actinobacteria, Actinomycetales, Propionibacteriaceae	0.0000	0.0000	0.0007	0.0004	0.0002	0.0002	0.0000	0.0000	1.00	0.87	1.00	1.00
Actinobacteria, Actinobacteria, Bifidobacteriales, Bifidobacteriaceae	5.4790	1.5724	10.2765	2.6920	6.3311	1.2241	9.6698	2.1906	1.00	1.00	0.74	1.00
Actinobacteria, Actinobacteria, Coriobacteriales, Coriobacteriaceae	0.2569	0.0629	0.4381	0.0649	0.3985	0.1183	0.4455	0.1057	1.00	0.91	0.40	1.00
Bacteroidetes, Other class, Other order, Other family	0.0398	0.0188	0.0246	0.0139	0.0065	0.0052	0.0002	0.0002	1.00	0.87	1.00	1.00
Bacteroidetes, Bacteroidia, Bacteroidales, Other family	0.4347	0.2253	0.2761	0.2203	0.0095	0.0072	0.0137	0.0137	1.00	0.87	1.00	1.00
Bacteroidetes, Bacteroidia, Bacteroidales, Bacteroidaceae	16.9896	4.4885	3.7743	0.8389	11.7978	3.5630	8.9581	3.1323	1.00	0.87	0.12	1.00
Bacteroidetes, Bacteroidia, Bacteroidales, Porphyromonadaceae	2.6905	0.8371	0.7105	0.2591	1.2856	0.2969	1.3994	0.4674	1.00	0.87	0.74	1.00
Bacteroidetes, Bacteroidia, Bacteroidales, Prevotellaceae	2.9752	1.5143	1.1329	0.7845	1.7609	0.8926	0.3886	0.1533	1.00	0.92	1.00	1.00
Bacteroidetes, Bacteroidia, Bacteroidales, Rikenellaceae	0.5964	0.1730	0.3051	0.1546	0.4632	0.2329	0.2936	0.0979	1.00	0.87	0.74	1.00
Candidatus Saccharibacteria, Other class, Other order, Other family	0.0058	0.0016	0.0099	0.0026	0.0066	0.0020	0.0083	0.0035	1.00	0.87	1.00	1.00
Cyanobacteria/Chloroplast, Chloroplast, Other order, Other family	0.0038	0.0019	0.0068	0.0037	0.0054	0.0034	0.0021	0.0012	1.00	0.87	1.00	1.00
Cyanobacteria/Chloroplast, Cyanobacteria, Other order, Other family	0.0000	0.0000	0.0000	0.0000	0.0006	0.0006	0.0000	0.0000	1.00	-	-	1.00
Firmicutes, Other class, Other order, Other family	0.1680	0.0650	0.1194	0.0336	0.2677	0.1759	0.2781	0.1460	1.00	1.00	1.00	1.00
Firmicutes, Bacilli, Other order, Other family	0.0000	0.0000	0.0006	0.0006	0.0000	0.0000	0.0004	0.0004	-	1.00	1.00	1.00
Firmicutes, Bacilli, Bacillales, Bacillaceae 1	0.0115	0.0056	0.0087	0.0061	0.0000	0.0000	0.0136	0.0098	0.74	0.87	1.00	1.00
Firmicutes, Bacilli, Bacillales, Bacillales_Incertae Sedis XI	0.0202	0.0067	0.0639	0.0422	0.0549	0.0294	0.0311	0.0086	1.00	0.87	1.00	1.00
Firmicutes, Bacilli, Bacillales, Listeriaceae	0.0000	0.0000	0.0000	0.0000	0.1083	0.1083	0.0000	0.0000	1.00	-	-	1.00
Firmicutes, Bacilli, Bacillales, Paenibacillaceae 1	0.0000	0.0000	0.0000	0.0000	0.0005	0.0005	0.0000	0.0000	1.00	-	-	1.00
Firmicutes, Bacilli, Bacillales, Staphylococcaceae	0.0022	0.0015	0.0015	0.0010	0.0043	0.0037	0.0008	0.0006	1.00	1.00	1.00	1.00
Firmicutes, Bacilli, Lactobacillales, Other family	0.0007	0.0005	0.0006	0.0004	0.0003	0.0003	0.0011	0.0008	1.00	1.00	1.00	1.00
Firmicutes, Bacilli, Lactobacillales, Aerococcaceae	0.0021	0.0013	0.0097	0.0052	0.0004	0.0004	0.0003	0.0003	1.00	0.87	1.00	1.00
Firmicutes, Bacilli, Lactobacillales, Carnobacteriaceae	0.0176	0.0053	0.0133	0.0054	0.2348	0.2154	0.0169	0.0072	1.00	1.00	1.00	1.00
Firmicutes, Bacilli, Lactobacillales, Enterococcaceae	0.3186	0.1868	0.4910	0.2445	0.0234	0.0113	0.0310	0.0211	1.00	0.87	1.00	1.00
Firmicutes, Bacilli, Lactobacillales, Lactobacillaceae	0.5160	0.2438	1.4652	0.7793	0.2481	0.1928	0.1445	0.0982	1.00	0.95	1.00	1.00
Firmicutes, Bacilli, Lactobacillales, Leuconostocaceae	0.0007	0.0004	0.0009	0.0006	0.0179	0.0148	0.0020	0.0010	1.00	0.87	1.00	1.00
Firmicutes, Bacilli, Lactobacillales, Streptococcaceae	2.2153	0.8105	9.0832	2.7580	5.7006	3.8051	6.5487	1.7114	1.00	0.91	0.07	1.00
Firmicutes, Clostridia, Other order, Other family	0.0083	0.0065	0.0028	0.0028	0.0188	0.0166	0.0186	0.0177	1.00	0.87	1.00	1.00
Firmicutes, Clostridia, Clostridiales, Other family	1.2237	0.4949	0.7063	0.2012	0.5991	0.2854	0.6487	0.3270	1.00	0.87	1.00	1.00
Firmicutes, Clostridia, Clostridiales, Catabacteriaceae	0.0025	0.0014	0.0086	0.0048	0.0024	0.0013	0.0008	0.0006	1.00	0.87	1.00	1.00
Firmicutes, Clostridia, Clostridiales, Christensenellaceae	0.0007	0.0007	0.0037	0.0037	0.0054	0.0034	0.0064	0.0040	1.00	0.87	1.00	1.00
Firmicutes, Clostridia, Clostridiales, Clostridiaceae 1	0.1988	0.0932	0.8478	0.7834	0.0421	0.0174	0.0706	0.0264	1.00	1.00	1.00	1.00
Firmicutes, Clostridia, Clostridiales, Clostridiales_Incertae Sedis XI	0.0062	0.0025	0.0487	0.0279	0.0055	0.0016	0.0046	0.0021	1.00	0.87	1.000	1.00
Firmicutes, Clostridia, Clostridiales, Clostridiales_Incertae Sedis XIII	0.2223	0.0925	0.3757	0.1130	0.3636	0.1129	0.3925	0.1579	1.00	1.00	1.00	1.00
Firmicutes, Clostridia, Clostridiales, Eubacteriaceae	0.0912	0.0664	0.1790	0.1082	0.0928	0.0396	0.0873	0.0299	1.00	1.00	1.00	1.00
Firmicutes, Clostridia, Clostridiales, Lachnospiraceae	38.9476	4.2941	45.9422	3.0268	41.9738	3.5312	44.4537	4.4503	1.00	1.00	0.75	1.00
Firmicutes, Clostridia, Clostridiales, Peptoniphilaceae	0.0037	0.0018	0.0048	0.0025	0.0019	0.0010	0.0010	0.0007	1.00	0.87	1.00	1.00
Firmicutes, Clostridia, Clostridiales, Peptostreptococcaceae	2.3278	0.9468	1.8649	0.6481	1.3918	0.3981	3.1623	1.4492	1.00	0.87	1.00	1.00
Firmicutes, Clostridia, Clostridiales, Ruminococcaceae	12.2286	2.2596	13.7721	2.6008	14.7720	2.7706	11.7578	2.4726	1.00	1.00	1.00	1.00
Firmicutes, Erysipelotrichia, Erysipelotrichales, Erysipelotrichaceae	4.4262	1.3508	3.9570	0.7132	4.2730	1.1484	6.4483	1.5434	1.00	0.87	1.00	1.00
Firmicutes, Negativicutes, Selenomonadales, Acidaminococcaceae	0.2982	0.1100	0.0605	0.0303	0.3048	0.0908	0.2365	0.1398	1.00	0.87	0.95	1.00
Firmicutes, Negativicutes, Selenomonadales, Veillonellaceae	3.1700	1.9099	0.8635	0.4771	5.1832	2.1909	2.4687	0.9032	1.00	0.87	0.74	1.00
Fusobacteria, Fusobacteriia, Fusobacteriales, Fusobacteriaceae	1.1078	0.8079	0.0560	0.0414	0.5837	0.3067	0.7442	0.6998	1.00	1.00	0.79	1.00
Fusobacteria, Fusobacteriia, Fusobacteriales, Leptotrichiaceae	0.0006	0.0006	0.0000	0.0000	0.0000	0.0000	0.0000	0.0000	1.00	-	1.00	-

Supplementary Table S6. Continued

Family	*Bifal*+Arg				Placebo				Difference (*p*-value)			
	Week 0		Week 12		Week 0		Week 12		*Bifal*+Arg VS. Placebo		Week 0 VS. Week 12	
	Mean	SEM	Mean	SEM	Mean	SEM	Mean	SEM	Week 0	Week 12	*Bifal*+Arg	Placebo
Lentisphaerae, Lentisphaeria, Victivallales, Victivallaceae	0.0012	0.0012	0.0000	0.0000	0.0003	0.0003	0.0000	0.0000	1.00	-	1.00	1.00
Proteobacteria, Other class, Other order, Other family	0.0010	0.0010	0.0013	0.0013	0.0004	0.0004	0.0000	0.0000	-	1.00	1.00	1.00
Proteobacteria, Alphaproteobacteria, Other order, Other family	0.0003	0.0003	0.0040	0.0040	0.0000	0.0000	0.0000	0.0000	1.00	1.00	1.00	-
Proteobacteria, Alphaproteobacteria, Caulobacterales, Caulobacteraceae	0.0003	0.0003	0.0000	0.0000	0.0000	0.0000	0.0004	0.0004	1.00	0.87	1.00	1.00
Proteobacteria, Alphaproteobacteria, Rhizobiales, Methylobacteriaceae	0.0000	0.0000	0.0000	0.0000	0.0000	0.0000	0.0003	0.0003	-	0.87	-	1.00
Proteobacteria, Alphaproteobacteria, Sphingomonadales, Sphingomonadaceae	0.0000	0.0000	0.0003	0.0003	0.0000	0.0000	0.0000	0.0000	-	1.00	1.00	-
Proteobacteria, Betaproteobacteria, Other order, Other family	0.0119	0.0090	0.0003	0.0003	0.0386	0.0373	0.0029	0.0022	1.00	0.87	0.75	1.00
Proteobacteria, Betaproteobacteria, Burkholderiales, Comamonadaceae	0.0000	0.0000	0.0003	0.0003	0.0000	0.0000	0.0000	0.0000	-	1.00	1.00	-
Proteobacteria, Betaproteobacteria, Burkholderiales, Oxalobacteraceae	0.0003	0.0003	0.0007	0.0007	0.0036	0.0036	0.0137	0.0137	1.00	1.00	1.00	1.00
Proteobacteria, Betaproteobacteria, Burkholderiales, Sutterellaceae	0.4359	0.1995	0.2802	0.1304	0.2013	0.1476	0.0894	0.0475	1.00	0.87	1.00	1.00
Proteobacteria, Deltaproteobacteria, Desulfovibrionales, Other family	0.0003	0.0003	0.0008	0.0008	0.0011	0.0011	0.0007	0.0007	1.00	1.00	1.00	1.00
Proteobacteria, Deltaproteobacteria, Desulfovibrionales, Desulfovibrionaceae	0.0328	0.0130	0.0117	0.0082	0.0629	0.0316	0.0221	0.0127	1.00	0.92	0.74	1.00
Proteobacteria, Epsilonproteobacteria, Campylobacterales, Campylobacteraceae	0.0098	0.0098	0.0000	0.0000	0.0000	0.0000	0.0000	0.0000	1.00	-	1.00	-
Proteobacteria, Epsilonproteobacteria, Campylobacterales, Helicobacteraceae	0.0000	0.0000	0.0000	0.0000	0.0003	0.0003	0.0000	0.0000	1.00	-	-	1.00
Proteobacteria, Gammaproteobacteria, Other order, Other family	0.0000	0.0000	0.0003	0.0003	0.0000	0.0000	0.0000	0.0000	-	1.00	-	-
Proteobacteria, Gammaproteobacteria, Enterobacteriales, Enterobacteriaceae	1.9746	0.6232	2.1152	0.9995	0.3964	0.1513	0.2160	0.0773	0.74	0.87	1.00	1.00
Proteobacteria, Gammaproteobacteria, Pasteurellales, Pasteurellaceae	0.0156	0.0062	0.0267	0.0119	0.0627	0.0289	0.0399	0.0250	1.00	1.00	1.00	1.00
Proteobacteria, Gammaproteobacteria, Pseudomonadales, Pseudomonadaceae	0.0002	0.0002	0.0000	0.0000	0.0002	0.0002	0.0000	0.0000	1.00	-	1.00	1.00
Proteobacteria, Gammaproteobacteria, Xanthomonadales, Xanthomonadaceae	0.0000	0.0000	0.0000	0.0000	0.0000	0.0000	0.0003	0.0003	1.00	0.87	-	-
Spirochaetes, Spirochaetia, Spirochaetales, Brachyspiraceae	0.0105	0.0105	0.0033	0.0033	0.0084	0.0080	0.0737	0.0645	1.00	0.87	1.00	1.00
Synergistetes, Synergistia, Synergistales, Synergistaceae	0.0004	0.0004	0.0050	0.0050	0.0002	0.0002	0.0007	0.0005	1.00	1.00	1.00	1.00
Verrucomicrobia, Verrucomicrobiae, Verrucomicrobiales, Verrucomicrobiaceae	0.0280	0.0185	0.0521	0.0319	0.0062	0.0058	0.0065	0.0065	1.00	0.87	1.00	1.00

Comparisons between the *Bifal*+Arg YG and placebo groups were tested using Mann-Whitney *U* tests. Comparisons at week 0 and at week 12 were tested using Wilcoxon signed rank tests. *p*-values were adjusted using the Benjamini and Hochberg method [1].

References

1. Benjamini, Y.; Hochberg, Y. Controlling the false discovery rate: a practical and powerful approach to multiple testing. *J. R. Statist. Soc. B (Methodological)* **1995**, 289-300.

Supplementary Table S7. Effect of consumption of yogurt containing *B. animalis* subsp. *lactis* and Arg (*Bifal*+Arg YG) on fecal microbiota composition (genus level)

	Bifal+Arg				Placebo				Difference (*p*-value)			
	Week 0		Week 12		Week 0		Week 12		*Bifal*+Arg VS. Placebo		Week 0 VS. Week 12	
Genus	Mean	SEM	Mean	SEM	Mean	SEM	Mean	SEM	Week 0	Week 12	*Bifal*+Arg	Placebo
Other phylum, Other class, Other order, Other family, Other genus	0.4468	0.1197	0.5738	0.2846	0.8376	0.5371	0.7690	0.3498	1.00	1.00	1.00	1.00
Actinobacteria, Actinobacteria, Other order, Other family, Other genus	0.0000	0.0000	0.0000	0.0000	0.0000	0.0000	0.0003	0.0003	-	1.00	-	1.00
Actinobacteria, Actinobacteria, Acidimicrobiales, Other family, Other genus	0.0006	0.0006	0.0000	0.0000	0.0000	0.0000	0.0000	0.0000	1.00	-	1.00	-
Actinobacteria, Actinobacteria, Actinomycetales, Other family, Other genus	0.0014	0.0008	0.0003	0.0003	0.0014	0.0006	0.0013	0.0006	1.00	1.00	1.00	1.00
Actinobacteria, Actinobacteria, Actinomycetales, Actinomycetaceae, *Actinomyces*	0.0141	0.0020	0.0115	0.0031	0.0272	0.0146	0.0059	0.0023	1.00	1.00	1.00	1.00
Actinobacteria, Actinobacteria, Actinomycetales, Actinomycetaceae, *Trueperella*	0.0000	0.0000	0.0000	0.0000	0.0003	0.0003	0.0017	0.0017	1.00	1.00	-	1.00
Actinobacteria, Actinobacteria, Actinomycetales, Corynebacteriaceae, *Corynebacterium*	0.0018	0.0012	0.0017	0.0010	0.0003	0.0003	0.0000	0.0000	1.00	1.00	1.00	1.00
Actinobacteria, Actinobacteria, Actinomycetales, Dermabacteraceae, *Brachybacterium*	0.0000	0.0000	0.0004	0.0004	0.0000	0.0000	0.0000	0.0000	-	1.00	1.00	-
Actinobacteria, Actinobacteria, Actinomycetales, Micrococcaceae, *Kocuria*	0.0000	0.0000	0.0000	0.0000	0.0000	0.0000	0.0008	0.0008	-	1.00	-	1.00
Actinobacteria, Actinobacteria, Actinomycetales, Micrococcaceae, *Rothia*	0.0033	0.0012	0.0030	0.0015	0.0099	0.0074	0.0048	0.0022	1.00	1.00	1.00	1.00
Actinobacteria, Actinobacteria, Actinomycetales, Propionibacteriaceae, Other genus	0.0000	0.0000	0.0003	0.0003	0.0000	0.0000	0.0000	0.0000	1.00	1.00	1.00	-
Actinobacteria, Actinobacteria, Actinomycetales, Propionibacteriaceae, *Propionibacterium*	0.0000	0.0000	0.0003	0.0003	0.0002	0.0002	0.0000	0.0000	1.00	1.00	1.00	1.00
Actinobacteria, Actinobacteria, Bifidobacteriales, Bifidobacteriaceae, Other genus	0.0015	0.0012	0.0024	0.0009	0.0022	0.0011	0.0013	0.0009	1.00	1.00	1.00	1.00
Actinobacteria, Actinobacteria, Bifidobacteriales, Bifidobacteriaceae, *Bifidobacterium*	5.4775	1.5724	10.2740	2.6919	6.3289	1.2233	9.6681	2.1898	1.00	1.00	1.00	1.00
Actinobacteria, Actinobacteria, Bifidobacteriales, Bifidobacteriaceae, *Gardnerella*	0.0000	0.0000	0.0000	0.0000	0.0000	0.0000	0.0005	0.0005	-	1.00	-	1.00
Actinobacteria, Actinobacteria, Coriobacteriales, Coriobacteriaceae, Other genus	0.0593	0.0222	0.1060	0.0496	0.1886	0.0810	0.1908	0.0853	1.00	1.00	1.00	1.00
Actinobacteria, Actinobacteria, Coriobacteriales, Coriobacteriaceae, *Atopobium*	0.0011	0.0011	0.0010	0.0005	0.0046	0.0037	0.0005	0.0003	1.00	1.00	1.00	1.00
Actinobacteria, Actinobacteria, Coriobacteriales, Coriobacteriaceae, *Collinsella*	0.0427	0.0158	0.0830	0.0295	0.0787	0.0376	0.0932	0.0262	1.00	1.00	1.00	1.00
Actinobacteria, Actinobacteria, Coriobacteriales, Coriobacteriaceae, *Eggerthella*	0.0983	0.0405	0.1685	0.0559	0.0758	0.0222	0.0780	0.0242	1.00	1.00	1.00	1.00
Actinobacteria, Actinobacteria, Coriobacteriales, Coriobacteriaceae, *Enterorhabdus*	0.0000	0.0000	0.0000	0.0000	0.0000	0.0000	0.0003	0.0003	-	1.00	-	1.00
Actinobacteria, Actinobacteria, Coriobacteriales, Coriobacteriaceae, *Gordonibacter*	0.0272	0.0151	0.0232	0.0072	0.0201	0.0093	0.0381	0.0123	1.00	1.00	1.00	1.00
Actinobacteria, Actinobacteria, Coriobacteriales, Coriobacteriaceae, *Olsenella*	0.0020	0.0018	0.0017	0.0017	0.0051	0.0038	0.0073	0.0069	1.00	1.00	1.00	1.00
Actinobacteria, Actinobacteria, Coriobacteriales, Coriobacteriaceae, *Paraeggerthella*	0.0000	0.0000	0.0005	0.0005	0.0000	0.0000	0.0000	0.0000	-	1.00	1.00	-
Actinobacteria, Actinobacteria, Coriobacteriales, Coriobacteriaceae, *Senegalimassilia*	0.0039	0.0036	0.0348	0.0232	0.0252	0.0153	0.0303	0.0189	1.00	1.00	1.00	1.00
Actinobacteria, Actinobacteria, Coriobacteriales, Coriobacteriaceae, *Slackia*	0.0224	0.0206	0.0194	0.0194	0.0004	0.0004	0.0068	0.0068	1.00	1.00	1.00	1.00
Bacteroidetes, Other class, Other order, Other family, Other genus	0.0398	0.0188	0.0246	0.0139	0.0065	0.0052	0.0002	0.0002	1.00	1.00	1.00	1.00
Bacteroidetes, Bacteroidia, Bacteroidales, Other family, Other genus	0.4347	0.2253	0.2761	0.2203	0.0095	0.0072	0.0137	0.0137	1.00	1.00	1.00	1.00
Bacteroidetes, Bacteroidia, Bacteroidales, Bacteroidaceae, *Bacteroides*	16.9896	4.4885	3.7743	0.8389	11.7978	3.5630	8.9581	3.1323	1.00	1.00	0.31	1.00
Bacteroidetes, Bacteroidia, Bacteroidales, Porphyromonadaceae, Other genus	0.0009	0.0007	0.0007	0.0004	0.0075	0.0075	0.0000	0.0000	1.00	1.00	1.00	1.00
Bacteroidetes, Bacteroidia, Bacteroidales, Porphyromonadaceae, *Barnesiella*	0.0862	0.0413	0.0708	0.0360	0.1067	0.0459	0.0885	0.0467	1.00	1.00	1.00	1.00
Bacteroidetes, Bacteroidia, Bacteroidales, Porphyromonadaceae, Butyricimonas	0.0180	0.0073	0.0129	0.0086	0.0116	0.0046	0.0073	0.0056	1.00	1.00	1.00	1.00
Bacteroidetes, Bacteroidia, Bacteroidales, Porphyromonadaceae, *Coprobacter*	0.0193	0.0115	0.0036	0.0024	0.0171	0.0092	0.0088	0.0054	1.00	1.00	1.00	1.00
Bacteroidetes, Bacteroidia, Bacteroidales, Porphyromonadaceae, *Odoribacter*	0.0537	0.0235	0.0294	0.0139	0.0582	0.0256	0.0299	0.0128	1.00	1.00	1.00	1.00
Bacteroidetes, Bacteroidia, Bacteroidales, Porphyromonadaceae, *Parabacteroides*	2.5115	0.8297	0.5847	0.2343	1.0844	0.2744	1.2650	0.4568	1.00	1.00	1.00	1.00
Bacteroidetes, Bacteroidia, Bacteroidales, Porphyromonadaceae, *Porphyromonas*	0.0008	0.0006	0.0084	0.0084	0.0000	0.0000	0.0000	0.0000	1.00	1.00	1.00	-
Bacteroidetes, Bacteroidia, Bacteroidales, Prevotellaceae, Other genus	0.8334	0.4874	0.2561	0.1929	0.1983	0.0865	0.0576	0.0265	1.00	1.00	1.00	1.00
Bacteroidetes, Bacteroidia, Bacteroidales, Prevotellaceae, *Alloprevotella*	0.0006	0.0006	0.0000	0.0000	0.0000	0.0000	0.0000	0.0000	1.00	-	1.00	-
Bacteroidetes, Bacteroidia, Bacteroidales, Prevotellaceae, Paraprevotella	0.1717	0.1091	0.0939	0.0816	0.0780	0.0373	0.0549	0.0362	1.00	1.00	1.00	1.00
Bacteroidetes, Bacteroidia, Bacteroidales, Prevotellaceae, *Prevotella*	1.9695	1.0371	0.7830	0.5919	1.4846	0.8809	0.2761	0.1265	1.00	1.00	1.00	1.00
Bacteroidetes, Bacteroidia, Bacteroidales, Rikenellaceae, *Alistipes*	0.5964	0.1730	0.3051	0.1546	0.4632	0.2329	0.2936	0.0979	1.00	1.00	1.00	1.00

Supplementary Table S7. Continued

Genus	*Bifal*+Arg				Placebo				Difference (*p*-value)			
	Week 0		Week 12		Week 0		Week 12		*Bifal*+Arg VS. Placebo		Week 0 VS. Week 12	
	Mean	SEM	Mean	SEM	Mean	SEM	Mean	SEM	Week 0	Week 12	*Bifal*+Arg	Placebo
Candidatus Saccharibacteria, Other class, Other order, Other family, Other genus	0.0058	0.0016	0.0099	0.0026	0.0066	0.0020	0.0083	0.0035	1.00	1.00	1.00	1.00
Cyanobacteria/Chloroplast, Chloroplast, Other order, Other family, Other genus	0.0038	0.0019	0.0068	0.0037	0.0054	0.0034	0.0021	0.0012	1.00	1.00	1.00	1.00
Cyanobacteria/Chloroplast, Cyanobacteria, Other order, Other family, Other genus	0.0000	0.0000	0.0000	0.0000	0.0006	0.0006	0.0000	0.0000	1.00	-	-	1.00
Firmicutes, Other class, Other order, Other family, Other genus	0.1680	0.0650	0.1194	0.0336	0.2677	0.1759	0.2781	0.1460	1.00	1.00	1.00	1.00
Firmicutes, Bacilli, Other order, Other family, Other genus	0.0000	0.0000	0.0006	0.0006	0.0000	0.0000	0.0004	0.0004	-	1.00	1.00	1.00
Firmicutes, Bacilli, Bacillales, Bacillaceae 1, *Bacillus*	0.0115	0.0056	0.0087	0.0061	0.0000	0.0000	0.0136	0.0098	1.00	1.00	1.00	1.00
Firmicutes, Bacilli, Bacillales, Bacillales_Incertae Sedis XI, *Gemella*	0.0202	0.0067	0.0639	0.0422	0.0549	0.0294	0.0311	0.0086	1.00	1.00	1.00	1.00
Firmicutes, Bacilli, Bacillales, Listeriaceae, *Brochothrix*	0.0000	0.0000	0.0000	0.0000	0.1083	0.1083	0.0000	0.0000	1.00	-	-	1.00
Firmicutes, Bacilli, Bacillales, Paenibacillaceae 1, *Paenibacillus*	0.0000	0.0000	0.0000	0.0000	0.0005	0.0005	0.0000	0.0000	1.00	-	-	1.00
Firmicutes, Bacilli, Bacillales, Staphylococcaceae, *Staphylococcus*	0.0022	0.0015	0.0015	0.0010	0.0043	0.0037	0.0008	0.0006	1.00	1.00	1.00	1.00
Firmicutes, Bacilli, Lactobacillales, Other family, Other genus	0.0007	0.0005	0.0006	0.0004	0.0003	0.0003	0.0011	0.0008	1.00	1.00	1.00	1.00
Firmicutes, Bacilli, Lactobacillales, Aerococcaceae, *Abiotrophia*	0.0021	0.0013	0.0097	0.0052	0.0004	0.0004	0.0003	0.0003	1.00	1.00	1.00	1.00
Firmicutes, Bacilli, Lactobacillales, Carnobacteriaceae, *Atopostipes*	0.0000	0.0000	0.0000	0.0000	0.0004	0.0004	0.0000	0.0000	1.00	-	-	1.00
Firmicutes, Bacilli, Lactobacillales, Carnobacteriaceae, *Carnobacterium*	0.0006	0.0005	0.0000	0.0000	0.2053	0.2053	0.0000	0.0000	1.00	-	1.00	1.00
Firmicutes, Bacilli, Lactobacillales, Carnobacteriaceae, *Granulicatella*	0.0170	0.0054	0.0133	0.0054	0.0291	0.0117	0.0169	0.0072	1.00	1.00	1.00	1.00
Firmicutes, Bacilli, Lactobacillales, Enterococcaceae, Other genus	0.0004	0.0004	0.0000	0.0000	0.0000	0.0000	0.0000	0.0000	1.00	-	1.00	-
Firmicutes, Bacilli, Lactobacillales, Enterococcaceae, *Enterococcus*	0.3183	0.1865	0.4910	0.2445	0.0160	0.0094	0.0310	0.0211	1.00	1.00	1.00	1.00
Firmicutes, Bacilli, Lactobacillales, Enterococcaceae, *Vagococcus*	0.0000	0.0000	0.0000	0.0000	0.0075	0.0075	0.0000	0.0000	1.00	-	-	1.00
Firmicutes, Bacilli, Lactobacillales, Lactobacillaceae, *Lactobacillus*	0.4799	0.2454	1.4538	0.7806	0.2481	0.1928	0.1445	0.0982	1.00	1.00	1.00	1.00
Firmicutes, Bacilli, Lactobacillales, Lactobacillaceae, *Pediococcus*	0.0362	0.0362	0.0115	0.0115	0.0000	0.0000	0.0000	0.0000	1.00	1.00	1.00	-
Firmicutes, Bacilli, Lactobacillales, Leuconostocaceae, *Leuconostoc*	0.0007	0.0004	0.0009	0.0006	0.0179	0.0148	0.0020	0.0010	1.00	1.00	1.00	1.00
Firmicutes, Bacilli, Lactobacillales, Streptococcaceae, *Lactococcus*	0.0022	0.0015	0.0037	0.0028	0.0027	0.0013	0.0052	0.0029	1.00	1.00	1.00	1.00
Firmicutes, Bacilli, Lactobacillales, Streptococcaceae, *Streptococcus*	2.2131	0.8107	9.0795	2.7556	5.6980	3.8053	6.5435	1.7118	1.00	1.00	0.16	1.00
Firmicutes, Clostridia, Other order, Other family, Other genus	0.0083	0.0065	0.0028	0.0028	0.0188	0.0166	0.0186	0.0177	1.00	1.00	1.00	1.00
Firmicutes, Clostridia, Clostridiales, Other family, Other genus	1.2237	0.4949	0.7063	0.2012	0.5991	0.2854	0.6487	0.3270	1.00	1.00	1.00	1.00
Firmicutes, Clostridia, Clostridiales, Catabacteriaceae, *Catabacter*	0.0025	0.0014	0.0086	0.0048	0.0024	0.0013	0.0008	0.0006	1.00	1.00	1.00	1.00
Firmicutes, Clostridia, Clostridiales, Christensenellaceae, *Christensenella*	0.0007	0.0007	0.0037	0.0037	0.0054	0.0034	0.0064	0.0040	1.00	1.00	1.00	1.00
Firmicutes, Clostridia, Clostridiales, Clostridiaceae 1, Other genus	0.0009	0.0009	0.0010	0.0010	0.0005	0.0003	0.0000	0.0000	1.00	1.00	1.000	1.00
Firmicutes, Clostridia, Clostridiales, Clostridiaceae 1, *Clostridium sensu stricto*	0.1979	0.0931	0.8468	0.7824	0.0415	0.0173	0.0706	0.0264	1.00	1.00	1.00	1.00
Firmicutes, Clostridia, Clostridiales, Clostridiales_Incertae.Sedis.XIII, Other genus	0.0450	0.0199	0.1123	0.0354	0.0869	0.0351	0.0894	0.0732	1.00	1.00	1.00	1.00
Firmicutes, Clostridia, Clostridiales, Clostridiales_Incertae Sedis XI, *Anaerococcus*	0.0011	0.0006	0.0019	0.0019	0.0003	0.0003	0.0009	0.0005	1.00	1.00	1.00	1.00
Firmicutes, Clostridia, Clostridiales, Clostridiales_Incertae Sedis XI, *Ezakiella*	0.0000	0.0000	0.0000	0.0000	0.0003	0.0003	0.0000	0.0000	1.00	-	-	1.00
Firmicutes, Clostridia, Clostridiales, Clostridiales_Incertae Sedis XI, *Finegoldia*	0.0031	0.0020	0.0025	0.0011	0.0033	0.0012	0.0006	0.0006	1.00	1.00	1.00	1.00
Firmicutes, Clostridia, Clostridiales, Clostridiales_Incertae Sedis XI, *Murdochiella*	0.0006	0.0006	0.0003	0.0003	0.0000	0.0000	0.0000	0.0000	1.00	1.00	1.00	-
Firmicutes, Clostridia, Clostridiales, Clostridiales_Incertae Sedis XI, *Parvimonas*	0.0014	0.0006	0.0440	0.0261	0.0016	0.0006	0.0031	0.0021	1.00	1.00	1.00	1.00
Firmicutes, Clostridia, Clostridiales, Clostridiales_Incertae Sedis XIII, *Anaerovorax*	0.1770	0.0734	0.2631	0.0922	0.2766	0.0948	0.3030	0.0999	1.00	1.00	1.00	1.00
Firmicutes, Clostridia, Clostridiales, Clostridiales_Incertae Sedis XIII, *Mogibacterium*	0.0004	0.0004	0.0003	0.0003	0.0000	0.0000	0.0000	0.0000	1.00	1.00	1.00	-
Firmicutes, Clostridia, Clostridiales, Eubacteriaceae, *Anaerofustis*	0.0056	0.0027	0.0084	0.0030	0.0047	0.0015	0.0048	0.0024	1.00	1.00	1.00	1.00
Firmicutes, Clostridia, Clostridiales, Eubacteriaceae, *Eubacterium*	0.0855	0.0668	0.1706	0.1079	0.0539	0.0247	0.0689	0.0289	1.00	1.00	1.00	1.00
Firmicutes, Clostridia, Clostridiales, Eubacteriaceae, Other genus	0.0000	0.0000	0.0000	0.0000	0.0342	0.0342	0.0135	0.0135	1.00	1.00	-	1.00
Firmicutes, Clostridia, Clostridiales, Lachnospiraceae, Other genus	7.3112	0.6341	10.8910	2.0492	11.9779	2.2694	14.4892	3.4438	1.00	1.00	1.00	1.00
Firmicutes, Clostridia, Clostridiales, Lachnospiraceae, *Anaerostipes*	2.7832	0.7368	3.7958	0.9211	4.0548	1.2317	3.5647	1.0190	1.00	1.00	1.00	1.00
Firmicutes, Clostridia, Clostridiales, Lachnospiraceae, *Blautia*	12.7611	1.5752	16.3307	1.4928	10.1640	1.1359	10.7721	1.3842	1.00	1.00	1.00	1.00

Supplementary Table S7. Continued

Genus	*Bifal*+Arg				Placebo				Difference (*p*-value)			
	Week 0		Week 12		Week 0		Week 12		*Bifal*+Arg VS. Placebo		Week 0 VS. Week 12	
	Mean	SEM	Mean	SEM	Mean	SEM	Mean	SEM	Week 0	Week 12	*Bifal*+Arg	Placebo
Firmicutes, Clostridia, Clostridiales, Lachnospiraceae, *Clostridium XlVa*	1.2307	0.3544	0.4075	0.0841	1.5590	0.5594	1.0897	0.3746	1.00	1.00	1.00	1.00
Firmicutes, Clostridia, Clostridiales, Lachnospiraceae, *Clostridium XlVb*	0.0751	0.0405	0.1514	0.1201	0.1698	0.1260	0.0804	0.0373	1.00	1.00	1.00	1.00
Firmicutes, Clostridia, Clostridiales, Lachnospiraceae, *Coprococcus*	0.3295	0.2483	0.3058	0.2164	0.1148	0.0595	0.2482	0.1468	1.00	1.00	1.00	1.00
Firmicutes, Clostridia, Clostridiales, Lachnospiraceae, *Dorea*	0.7804	0.2861	0.9985	0.3143	1.1839	0.3064	1.4933	0.5007	1.00	1.00	1.00	1.00
Firmicutes, Clostridia, Clostridiales, Lachnospiraceae, *Eisenbergiella*	0.0496	0.0237	0.0880	0.0389	0.0340	0.0216	0.0383	0.0176	1.00	1.00	1.00	1.00
Firmicutes, Clostridia, Clostridiales, Lachnospiraceae, *Fusicatenibacter*	2.2636	0.7281	4.6474	1.6945	3.1636	0.8620	2.7943	0.9358	1.00	1.00	1.00	1.00
Firmicutes, Clostridia, Clostridiales, Lachnospiraceae, *Howardella*	0.0005	0.0005	0.0029	0.0029	0.0000	0.0000	0.0000	0.0000	1.00	1.00	1.00	-
Firmicutes, Clostridia, Clostridiales, Lachnospiraceae, *Hungatella*	0.0062	0.0044	0.0104	0.0054	0.0122	0.0078	0.0135	0.0056	1.00	1.00	1.00	1.00
Firmicutes, Clostridia, Clostridiales, Lachnospiraceae, *Lachnoanaerobaculum*	0.0000	0.0000	0.0003	0.0003	0.0000	0.0000	0.0006	0.0006	-	1.00	1.00	1.00
Firmicutes, Clostridia, Clostridiales, Lachnospiraceae, *Lachnospiracea_incertae_sedis*	7.6101	2.6875	6.6192	1.6844	7.0984	1.7810	7.5811	1.9140	1.00	1.00	1.00	1.00
Firmicutes, Clostridia, Clostridiales, Lachnospiraceae, *Lactonifactor*	0.0303	0.0259	0.0218	0.0123	0.0250	0.0126	0.0154	0.0083	1.00	1.00	1.00	1.00
Firmicutes, Clostridia, Clostridiales, Lachnospiraceae, *Murimonas*	0.0008	0.0008	0.0000	0.0000	0.0000	0.0000	0.0000	0.0000	1.00	-	1.00	-
Firmicutes, Clostridia, Clostridiales, Lachnospiraceae, *Oribacterium*	0.0005	0.0003	0.0016	0.0007	0.0003	0.0003	0.0018	0.0011	1.00	1.00	1.00	1.00
Firmicutes, Clostridia, Clostridiales, Lachnospiraceae, *Robinsoniella*	0.0000	0.0000	0.0027	0.0027	0.0000	0.0000	0.0000	0.0000	-	1.00	1.00	-
Firmicutes, Clostridia, Clostridiales, Lachnospiraceae, *Roseburia*	2.5363	1.2481	0.8731	0.2538	1.4126	0.4523	1.1089	0.3540	1.00	1.00	1.00	1.00
Firmicutes, Clostridia, Clostridiales, Lachnospiraceae, *Ruminococcus 2*	1.1781	0.4972	0.7942	0.3354	1.0035	0.2952	1.1621	0.5335	1.00	1.00	1.00	1.00
Firmicutes, Clostridia, Clostridiales, Lachnospiraceae, *Shuttleworthia*	0.0003	0.0003	0.0000	0.0000	0.0000	0.0000	0.0000	0.0000	1.00	-	1.00	-
Firmicutes, Clostridia, Clostridiales, Lachnospiraceae, *Syntrophococcus*	0.0000	0.0000	0.0000	0.0000	0.0000	0.0000	0.0002	0.0002	-	1.00	-	1.00
Firmicutes, Clostridia, Clostridiales, Peptoniphilaceae, *Peptoniphilus*	0.0037	0.0018	0.0048	0.0025	0.0019	0.0010	0.0010	0.0007	1.00	1.00	1.00	1.00
Firmicutes, Clostridia, Clostridiales, Peptostreptococcaceae, *Clostridium XI*	0.0485	0.0485	0.1287	0.1136	0.0000	0.0000	0.0000	0.0000	1.00	1.00	1.00	-
Firmicutes, Clostridia, Clostridiales, Peptostreptococcaceae, *Intestinibacter*	0.9636	0.4575	0.8757	0.4084	0.4938	0.2507	2.2227	1.2564	1.00	1.00	1.00	1.00
Firmicutes, Clostridia, Clostridiales, Peptostreptococcaceae, Other genus	0.0389	0.0143	0.0446	0.0143	0.0297	0.0108	0.0765	0.0300	1.00	1.00	1.00	1.00
Firmicutes, Clostridia, Clostridiales, Peptostreptococcaceae, *Peptostreptococcus*	0.0129	0.0052	0.0371	0.0232	0.0221	0.0120	0.0118	0.0066	1.00	1.00	1.00	1.00
Firmicutes, Clostridia, Clostridiales, Peptostreptococcaceae, *Romboutsia*	0.9731	0.4800	0.7349	0.3285	0.8060	0.2343	0.7686	0.2511	1.00	1.00	1.00	1.00
Firmicutes, Clostridia, Clostridiales, Peptostreptococcaceae, *Terrisporobacter*	0.2909	0.2736	0.0440	0.0263	0.0402	0.0307	0.0827	0.0390	1.00	1.00	1.00	1.00
Firmicutes, Clostridia, Clostridiales, Ruminococcaceae, *Anaerofilum*	0.0008	0.0005	0.0011	0.0011	0.0019	0.0012	0.0009	0.0006	1.00	1.00	1.00	1.00
Firmicutes, Clostridia, Clostridiales, Ruminococcaceae, *Anaerotruncus*	0.0093	0.0037	0.0024	0.0012	0.0053	0.0023	0.0045	0.0022	1.00	1.00	1.00	1.00
Firmicutes, Clostridia, Clostridiales, Ruminococcaceae, *Butyricicoccus*	0.4481	0.1065	0.8163	0.1817	0.7758	0.1715	0.8882	0.1289	1.00	1.00	1.00	1.00
Firmicutes, Clostridia, Clostridiales, Ruminococcaceae, *Clostridium IV*	0.1778	0.0838	0.0861	0.0308	0.2281	0.0853	0.1413	0.0577	1.00	1.00	1.00	1.00
Firmicutes, Clostridia, Clostridiales, Ruminococcaceae, *Faecalibacterium*	4.7356	1.0962	5.5405	1.5218	6.9576	1.8497	5.1520	1.7276	1.00	1.00	1.00	1.00
Firmicutes, Clostridia, Clostridiales, Ruminococcaceae, *Flavonifractor*	0.1321	0.0333	0.1981	0.0585	0.1351	0.0658	0.1335	0.0367	1.00	1.00	1.00	1.00
Firmicutes, Clostridia, Clostridiales, Ruminococcaceae, *Intestinimonas*	0.0063	0.0040	0.0242	0.0182	0.0019	0.0011	0.0057	0.0025	1.00	1.00	1.00	1.00
Firmicutes, Clostridia, Clostridiales, Ruminococcaceae, *Oscillibacter*	0.5850	0.4572	0.1534	0.0644	0.1781	0.0723	0.1437	0.0802	1.00	1.00	1.00	1.00
Firmicutes, Clostridia, Clostridiales, Ruminococcaceae, Other genus	5.4794	1.5143	6.3345	1.9010	6.1176	1.5179	4.9729	1.2200	1.00	1.00	1.00	1.00
Firmicutes, Clostridia, Clostridiales, Ruminococcaceae, *Pseudoflavonifractor*	0.0418	0.0167	0.0280	0.0107	0.0575	0.0201	0.0752	0.0292	1.00	1.00	1.00	1.00
Firmicutes, Clostridia, Clostridiales, Ruminococcaceae, *Ruminococcus*	0.5890	0.2094	0.5673	0.2639	0.2972	0.1361	0.2243	0.1159	1.00	1.00	1.00	1.00
Firmicutes, Clostridia, Clostridiales, Ruminococcaceae, *Sporobacter*	0.0003	0.0003	0.0000	0.0000	0.0000	0.0000	0.0002	0.0002	1.00	1.00	1.00	1.00
Firmicutes, Clostridia, Clostridiales, Ruminococcaceae, *Subdoligranulum*	0.0231	0.0067	0.0200	0.0096	0.0159	0.0034	0.0152	0.0037	1.00	1.00	1.00	1.00
Firmicutes, Erysipelotrichia, Erysipelotrichales, Erysipelotrichaceae, Other genus	1.6355	0.5786	1.7473	0.5765	0.6273	0.2037	1.4850	0.7422	1.00	1.00	1.00	1.00
Firmicutes, Erysipelotrichia, Erysipelotrichales, Erysipelotrichaceae, *Bulleidia*	0.0000	0.0000	0.0000	0.0000	0.0007	0.0007	0.0000	0.0000	1.00	-	-	1.00
Firmicutes, Erysipelotrichia, Erysipelotrichales, Erysipelotrichaceae, *Catenibacterium*	0.0537	0.0530	0.0225	0.0215	1.1799	0.9406	1.8419	1.3018	1.00	1.00	1.00	1.00
Firmicutes, Erysipelotrichia, Erysipelotrichales, Erysipelotrichaceae, *Clostridium XVIII*	1.1454	0.6376	1.0430	0.3120	0.7452	0.3217	1.2358	0.5301	1.00	1.00	1.00	1.00
Firmicutes, Erysipelotrichia, Erysipelotrichales, Erysipelotrichaceae, *Coprobacillus*	0.0169	0.0113	0.0482	0.0240	0.0104	0.0064	0.0030	0.0018	1.00	1.00	1.00	1.00

Supplementary Table S7. Continued

Genus	*Bifal*+Arg				Placebo				Difference (*p*-value)			
	Week 0		Week 12		Week 0		Week 12		*Bifal*+Arg VS. Placebo		Week 0 VS. Week 12	
	Mean	SEM	Mean	SEM	Mean	SEM	Mean	SEM	Week 0	Week 12	*Bifal*+Arg	Placebo
Firmicutes, Erysipelotrichia, Erysipelotrichales, Erysipelotrichaceae, *Erysipelotrichaceae_incertae_sedis*	0.9461	0.6509	0.6851	0.3180	0.1273	0.0443	0.1623	0.0763	1.00	1.00	1.00	1.00
Firmicutes, Erysipelotrichia, Erysipelotrichales, Erysipelotrichaceae, *Faecalicoccus*	0.0006	0.0006	0.0000	0.0000	0.0000	0.0000	0.0000	0.0000	1.00	-	1.00	-
Firmicutes, Erysipelotrichia, Erysipelotrichales, Erysipelotrichaceae, *Holdemanella*	0.3908	0.2719	0.1776	0.1366	1.0524	0.7122	1.5698	1.0899	1.00	1.00	1.00	1.00
Firmicutes, Erysipelotrichia, Erysipelotrichales, Erysipelotrichaceae, *Holdemania*	0.0094	0.0035	0.0188	0.0119	0.0166	0.0060	0.0242	0.0094	1.00	1.00	1.00	1.00
Firmicutes, Erysipelotrichia, Erysipelotrichales, Erysipelotrichaceae, *Solobacterium*	0.0042	0.0011	0.0181	0.0116	0.0101	0.0068	0.0048	0.0026	1.00	1.00	1.00	1.00
Firmicutes, Erysipelotrichia, Erysipelotrichales, Erysipelotrichaceae, *Turicibacter*	0.2237	0.0849	0.1964	0.1156	0.5031	0.2133	0.1216	0.0456	1.00	1.00	1.00	1.00
Firmicutes, Negativicutes, Selenomonadales, Acidaminococcaceae, Other genus	0.0360	0.0330	0.0054	0.0030	0.0190	0.0190	0.0039	0.0039	1.00	1.00	1.00	1.00
Firmicutes, Negativicutes, Selenomonadales, Acidaminococcaceae, *Acidaminococcus*	0.0600	0.0316	0.0363	0.0258	0.1234	0.0587	0.0449	0.0242	1.00	1.00	1.00	1.00
Firmicutes, Negativicutes, Selenomonadales, Acidaminococcaceae, *Phascolarctobacterium*	0.2022	0.0919	0.0188	0.0170	0.1624	0.0781	0.1876	0.1413	1.00	1.00	1.00	1.00
Firmicutes, Negativicutes, Selenomonadales, Veillonellaceae, Other genus	0.0622	0.0377	0.0407	0.0179	0.0214	0.0182	0.0075	0.0064	1.00	1.00	1.00	1.00
Firmicutes, Negativicutes, Selenomonadales, Veillonellaceae, *Allisonella*	0.0291	0.0162	0.0103	0.0043	0.0211	0.0088	0.0089	0.0049	1.00	1.00	1.00	1.00
Firmicutes, Negativicutes, Selenomonadales, Veillonellaceae, *Anaeroglobus*	0.0002	0.0002	0.0000	0.0000	0.0000	0.0000	0.0000	0.0000	1.00	-	1.00	-
Firmicutes, Negativicutes, Selenomonadales, Veillonellaceae, *Dialister*	0.0940	0.0476	0.0872	0.0785	0.4061	0.1921	0.2426	0.1666	1.00	1.00	1.00	1.00
Firmicutes, Negativicutes, Selenomonadales, Veillonellaceae, *Megamonas*	2.0648	1.7889	0.4636	0.4579	2.1429	1.5687	1.7080	0.8655	1.00	1.00	1.00	1.00
Firmicutes, Negativicutes, Selenomonadales, Veillonellaceae, *Megasphaera*	0.3333	0.1456	0.1172	0.0506	2.4914	1.6603	0.4229	0.1686	1.00	1.00	1.00	1.00
Firmicutes, Negativicutes, Selenomonadales, Veillonellaceae, *Mitsuokella*	0.2688	0.2414	0.0547	0.0426	0.0038	0.0024	0.0065	0.0049	1.00	1.00	1.00	1.00
Firmicutes, Negativicutes, Selenomonadales, Veillonellaceae, *Propionispira*	0.0004	0.0004	0.0000	0.0000	0.0000	0.0000	0.0000	0.0000	1.00	-	1.00	-
Firmicutes, Negativicutes, Selenomonadales, Veillonellaceae, *Veillonella*	0.3172	0.1586	0.0898	0.0412	0.0965	0.0447	0.0723	0.0323	1.00	1.00	1.00	1.00
Fusobacteria, Fusobacteriia, Fusobacteriales, Fusobacteriaceae, Other genus	0.0226	0.0222	0.0004	0.0004	0.0026	0.0023	0.0166	0.0166	1.00	1.00	1.00	1.00
Fusobacteria, Fusobacteriia, Fusobacteriales, Fusobacteriaceae, *Fusobacterium*	1.0852	0.7860	0.0557	0.0410	0.5811	0.3056	0.7276	0.6832	1.00	1.00	1.00	1.00
Fusobacteria, Fusobacteriia, Fusobacteriales, Leptotrichiaceae, *Sneathia*	0.0006	0.0006	0.0000	0.0000	0.0000	0.0000	0.0000	0.0000	1.00	-	1.00	-
Lentisphaerae, Lentisphaeria, Victivallales, Victivallaceae, *Victivallis*	0.0012	0.0012	0.0000	0.0000	0.0003	0.0003	0.0000	0.0000	1.00	-	1.00	1.00
Proteobacteria, Other class, Other order, Other family, Other genus	0.0010	0.0010	0.0013	0.0013	0.0004	0.0004	0.0000	0.0000	1.00	1.00	1.00	1.00
Proteobacteria, Alphaproteobacteria, Other order, Other family, Other genus	0.0003	0.0003	0.0040	0.0040	0.0000	0.0000	0.0000	0.0000	1.00	1.00	1.00	-
Proteobacteria, Alphaproteobacteria, Caulobacterales, Caulobacteraceae, *Brevundimonas*	0.0003	0.0003	0.0000	0.0000	0.0000	0.0000	0.0004	0.0004	1.00	1.00	1.00	1.00
Proteobacteria, Alphaproteobacteria, Rhizobiales, Methylobacteriaceae, *Methylobacterium*	0.0000	0.0000	0.0000	0.0000	0.0000	0.0000	0.0003	0.0003	-	1.00	-	1.00
Proteobacteria, Alphaproteobacteria, Sphingomonadales, Sphingomonadaceae, *Sphingomonas*	0.0000	0.0000	0.0003	0.0003	0.0000	0.0000	0.0000	0.0000	-	1.00	1.00	-
Proteobacteria, Betaproteobacteria, Other order, Other family, Other genus	0.0119	0.0090	0.0003	0.0003	0.0386	0.0373	0.0029	0.0022	1.00	1.00	1.00	1.00
Proteobacteria, Betaproteobacteria, Burkholderiales, Comamonadaceae, *Pelomonas*	0.0000	0.0000	0.0003	0.0003	0.0000	0.0000	0.0000	0.0000	-	1.00	1.00	-
Proteobacteria, Betaproteobacteria, Burkholderiales, Oxalobacteraceae, *Oxalobacter*	0.0003	0.0003	0.0007	0.0007	0.0036	0.0036	0.0137	0.0137	1.00	1.00	1.00	1.00
Proteobacteria, Betaproteobacteria, Burkholderiales, Sutterellaceae, *Parasutterella*	0.4212	0.2013	0.2782	0.1307	0.1933	0.1479	0.0796	0.0480	1.00	1.00	1.00	1.00
Proteobacteria, Betaproteobacteria, Burkholderiales, Sutterellaceae, *Sutterella*	0.0148	0.0076	0.0020	0.0017	0.0080	0.0041	0.0098	0.0074	1.00	1.00	1.00	1.00
Proteobacteria, Deltaproteobacteria, Desulfovibrionales, Other family, Other genus	0.0003	0.0003	0.0008	0.0008	0.0011	0.0011	0.0007	0.0007	1.00	1.00	1.00	1.00
Proteobacteria, Deltaproteobacteria, Desulfovibrionales, Desulfovibrionaceae, *Bilophila*	0.0222	0.0075	0.0114	0.0082	0.0258	0.0109	0.0084	0.0037	1.00	1.00	1.00	1.00
Proteobacteria, Deltaproteobacteria, Desulfovibrionales, Desulfovibrionaceae, *Desulfovibrio*	0.0106	0.0097	0.0003	0.0003	0.0343	0.0247	0.0125	0.0108	1.00	1.00	1.00	1.00
Proteobacteria, Deltaproteobacteria, Desulfovibrionales, Desulfovibrionaceae, Other genus	0.0000	0.0000	0.0000	0.0000	0.0028	0.0028	0.0012	0.0012	1.00	1.00	-	1.00
Proteobacteria, Epsilonproteobacteria, Campylobacterales, Campylobacteraceae, *Campylobacter*	0.0098	0.0098	0.0000	0.0000	0.0000	0.0000	0.0000	0.0000	1.00	-	1.00	-
Proteobacteria, Epsilonproteobacteria, Campylobacterales, Helicobacteraceae, *Helicobacter*	0.0000	0.0000	0.0000	0.0000	0.0003	0.0003	0.0000	0.0000	1.00	-	-	1.00
Proteobacteria, Gammaproteobacteria, Other order, Other family, Other genus	0.0000	0.0000	0.0003	0.0003	0.0000	0.0000	0.0000	0.0000	-	1.00	1.00	-
Proteobacteria, Gammaproteobacteria, Enterobacteriales, Enterobacteriaceae, Other genus	1.6197	0.5119	1.8257	0.9268	0.3001	0.1212	0.1590	0.0550	1.00	1.00	1.00	1.00
Proteobacteria, Gammaproteobacteria, Enterobacteriales, Enterobacteriaceae, *Citrobacter*	0.0897	0.0365	0.1612	0.0601	0.0264	0.0183	0.0121	0.0051	1.00	1.00	1.00	1.00
Proteobacteria, Gammaproteobacteria, Enterobacteriales, Enterobacteriaceae, *Cronobacter*	0.0025	0.0013	0.0045	0.0037	0.0003	0.0003	0.0000	0.0000	1.00	1.00	1.00	1.00

Supplementary Table S7. Continued

Genus	*Bifal*+Arg				Placebo				Difference (*p*-value)			
	Week 0		Week 12		Week 0		Week 12		*Bifal*+Arg VS. Placebo		Week 0 VS. Week 12	
	Mean	SEM	Mean	SEM	Mean	SEM	Mean	SEM	Week 0	Week 12	*Bifal*+Arg	Placebo
Proteobacteria, Gammaproteobacteria, Enterobacteriales, Enterobacteriaceae, *Edwardsiella*	0.0079	0.0079	0.0000	0.0000	0.0000	0.0000	0.0003	0.0003	1.00	1.00	1.00	1.00
Proteobacteria, Gammaproteobacteria, Enterobacteriales, Enterobacteriaceae, *Escherichia/Shigella*	0.2320	0.0854	0.1107	0.0590	0.0607	0.0274	0.0361	0.0227	1.00	1.00	1.00	1.00
Proteobacteria, Gammaproteobacteria, Enterobacteriales, Enterobacteriaceae, *Klebsiella*	0.0216	0.0102	0.0125	0.0078	0.0089	0.0045	0.0085	0.0055	1.00	1.00	1.00	1.00
Proteobacteria, Gammaproteobacteria, Enterobacteriales, Enterobacteriaceae, *Morganella*	0.0004	0.0004	0.0006	0.0004	0.0000	0.0000	0.0000	0.0000	1.00	1.00	1.00	-
Proteobacteria, Gammaproteobacteria, Enterobacteriales, Enterobacteriaceae, *Proteus*	0.0004	0.0004	0.0000	0.0000	0.0000	0.0000	0.0000	0.0000	1.00	-	1.00	-
Proteobacteria, Gammaproteobacteria, Enterobacteriales, Enterobacteriaceae, *Pseudocitrobacter*	0.0004	0.0004	0.0000	0.0000	0.0000	0.0000	0.0000	0.0000	1.00	-	1.00	-
Proteobacteria, Gammaproteobacteria, Pasteurellales, Pasteurellaceae, Other genus	0.0000	0.0000	0.0003	0.0003	0.0006	0.0004	0.0003	0.0003	1.00	1.00	1.00	1.00
Proteobacteria, Gammaproteobacteria, Pasteurellales, Pasteurellaceae, *Haemophilus*	0.0156	0.0062	0.0264	0.0116	0.0621	0.0287	0.0396	0.0249	1.00	1.00	1.00	1.00
Proteobacteria, Gammaproteobacteria, Pseudomonadales, Pseudomonadaceae, Other genus	0.0000	0.0000	0.0000	0.0000	0.0002	0.0002	0.0000	0.0000	1.00	-	-	1.00
Proteobacteria, Gammaproteobacteria, Pseudomonadales, Pseudomonadaceae, *Pseudomonas*	0.0002	0.0002	0.0000	0.0000	0.0000	0.0000	0.0000	0.0000	1.00	-	1.00	-
Proteobacteria, Gammaproteobacteria, Xanthomonadales, Xanthomonadaceae, *Vulcaniibacterium*	0.0000	0.0000	0.0000	0.0000	0.0000	0.0000	0.0003	0.0003	-	1.00	-	1.00
Spirochaetes, Spirochaetia, Spirochaetales, Brachyspiraceae, *Brachyspira*	0.0105	0.0105	0.0033	0.0033	0.0084	0.0080	0.0737	0.0645	1.00	1.00	1.00	1.00
Synergistetes, Synergistia, Synergistales, Synergistaceae, *Cloacibacillus*	0.0004	0.0004	0.0050	0.0050	0.0002	0.0002	0.0007	0.0005	1.00	1.00	1.00	1.00
Verrucomicrobia, Verrucomicrobiae, Verrucomicrobiales, Verrucomicrobiaceae, *Akkermansia*	0.0280	0.0185	0.0521	0.0319	0.0062	0.0058	0.0065	0.0065	1.00	1.00	1.00	1.00

Comparisons between the *Bifal*+Arg YG and placebo groups were tested using Mann-Whitney *U* tests. Comparisons at week 0 and at week 12 were tested using Wilcoxon signed rank tests. *p*-values were adjusted using the Benjamini and Hochberg method [1].

References

1. Benjamini, Y.; Hochberg, Y. Controlling the false discovery rate: a practical and powerful approach to multiple testing. *J. R. Statist. Soc. B (Methodological)* **1995**, 289-300.
